# Supplementary material for: Understanding variation in unplanned admissions of people aged 85 and over: a systems-based approach
Source: BMJ Open. 2019 Jul 9;9(7):e026405. doi: 10.1136/bmjopen-2018-026405 (PMC6615796; doi:10.1136/bmjopen-2018-026405)
Supplement: Supplementary data [file bmjopen-2018-026405supp001.pdf]

Appendix 1: detailed topic guide

Unplanned admissions in the area: priorities, levels and changes Ask interviewee to give a reaction to the data presented to them giving the rates of unplanned admissions of people 85+ in their area during 2005–9.

Historical perspective from 2005 onwards.

Possible questions and prompts:

(a) Were you aware of the rate of unplanned admissions to hospital of people 85+ in your area? (b) Did you know if your rates were increasing decreasing or staying the same? – What would you say were the reasons for that?

(c) Are the rates of unplanned admissions to acute hospital of people 85+ an issue in your organisation? – Do they need to be reduced?

(d) Do you collect/collate any local data on unplanned admissions and if so can we see the reports/have access to the data?

Whole system characteristics, configuration, monitoring and changes over time What are the important elements/components which you believe have the most effect on the rates of unplanned admissions of people 85+ in your area?

Possible prompts (could use star model to illustrate):

(a) Policies. – Procedures, service delivery, working practices.

(b) People, staffing, leadership, behaviour. (c) Finance and budgets. (d) Infrastructure, geography, buildings, IT. (e) Partner organisations. (f) Shared vision.

Possible questions:

(a) What characteristics are associated with changes in unplanned admissions of people 85+? (b) What changes have you made as a result of the increasing number of people over 85? (c) What changes do you think you need to make to reduce unplanned admissions of people 85+? (d) What changes are planned to reduce unplanned admissions of people 85+? (e) What have been the historical drivers of change? – Leadership. – Finance. – Targets.

(f) When making changes what have been the main challenges? (g) What have been the main outcomes of change? – Did it achieve what was hoped for?

(h) Reasons for success or failure of changes.

Policy on unplanned admissions

(a) How have/do policies in your organisation affect unplanned admissions of people aged 85+? – Historical perspective as well as current. – E.g. A&E, ED, Primary Care, out-of-hours services, Ambulance, Staffing.

(b) What have been/are your organisation’s policies around unplanned admissions? – Historical perspective as well as current.

(c) Have policies around unplanned admissions been linked/coherent with the policies of other organisations in your area? – Historical perspective as well as current.

(d) What policy changes have you made as a result of the increasing number of people over 85? (e) Have there been/are there clear strategies/goals within your organisation around unplanned admissions of people 85+? (f) Please can you supply policy documentation on unplanned admissions of people 85+ from 2005 to the present?

Budgets/finance/commissioning of emergency care

(a) Is there a specific budget for unplanned/emergency care for people aged 85+/older people? – Historically and currently.

(b) How much is spent on emergency care for people aged 85+? – Historically and currently.

(c) How much is spent on older people compared with other areas? – Historically and currently.

(d) What are the commissioning arrangements for unplanned admissions (of people 85+) in your area? – CCGs. – PCTs. – Involvement of secondary care.

(e) How have these arrangements changed since 2005? (f) What part have financial imperatives (cost/resources) played in commissioning services for unplanned admissions? (g) What are the incentives in commissioning which affect unplanned admissions?

Partnership working between acute and community health and social care

(a) How have acute and community health and social care worked together in the area to tackle unplanned admissions and care of older people? (b) Has there been a whole systems approach to planning care or do all the organisations commission, plan and deliver service separately? E.g. – Cooperative inter organisational networks. – Sharing information and communication. – Shared protocols and procedures. – Shared policy on unplanned admissions – linked/coherent?

(c) Have there been any integrated services or is this planned? (d) What have been the incentives to a whole systems approach? (e) What have been the challenges to a whole systems approach and how does cooperation/competition pose challenges? (f) Do the health- and social care organisations in your area have clearly defined boundaries? – What are the boundaries? – Are boundaries a problem? – Different measurement criteria for success? – Different organisational cultures? – Different regulation?

(g) Outcomes of partnership/integrated planning/services? – Successes and challenges. – Reasons for success or failure. – Obtain copies of any partnership agreements.

People, leadership, managerial and clinical relationships, staffing

(a) Who has led policy and/or changes affecting unplanned admissions of people over 85 in this area? – Organisational leadership.

(b) Have there been any professional tensions, allegiances and hierarchies which affect unplanned admissions in this area? (c) Have there been any staff satisfaction surveys carried out in your organisation? – Can we have report?

(d) Have job planning and staffing changes had an impact on unplanned admissions? (e) What impact has training had on unplanned admissions?

Infrastructure and contextual factors influencing unplanned admissions of 85+

(a) Have there been any local infrastructure or contextual factors or pressures influencing unplanned admissions of 85+ in this area? Possible prompts: – Geography. – Buildings/facilities. – Technology/IT systems. – Transport – ambulance services. – Environmental conditions. – Local pressures.

Procedures/service delivery

(a) Are there any issues around service delivery in your area which have affected unplanned admissions of people 85+? Possible prompts: – Who delivers urgent care services in the acute sector? – Who delivers urgent care services in the community sector? – Commissioning arrangements. – Pathways of care. – Access points for urgent and emergency care. – Admission pathways. – Management of acute care. – ED configuration. – Integrated care. – Access to and responsiveness of social care. – Management of chronic conditions. – Out-of-hours provision in the community. – Ambulance service. – Discharge procedures. – IT systems (Dashboard, NHS Pathways, discharge monitoring systems, etc.). – Governance systems (emergency care networks, joint governance frameworks).

Interventions, outcomes and evaluation

(a) What interventions in the whole system have affected or changed the levels of unplanned admissions? – Intermediate care. – Integrated care. – Reablement – End of life care. – Residential/nursing home care. – Community matrons. – Alternatives to acute admission. – Discharge initiatives. – Social care initiatives. – Chronic conditions management. – Geriatrician assessment/outreach. – Hospital outreach. – Hospice care/outreach.

(b) Who delivers these services? (c) What has been the effect of these interventions on the whole system of care? (d) What is the relationship between interventions? (e) How available and accessible are alternatives to unplanned admission? – E.g. primary and social care services.

(f) Have the outcomes of the interventions been measured or evaluated? (g) What are the reasons for the success of these interventions? (h) What are the reasons for the failure of these interventions? (i) Can I have copies of any internal evaluations?

Recommendations for reducing emergency admissions

(a) What changes have been made/need to be made to reduce unplanned admissions? – Strategy. – Organisational. – Partnership. – Commissioning. – Policy. – Service delivery. – Leadership/staffing.

(b) What lessons have you learnt in trying to reduce unplanned admissions for those 85+? – What are the challenges? – What have been the successes?

PPI involvement

(a) What PPI involvement do you have in your organisation in this area? (b) What PPI involvement do you have in policy making in this area? (c) What PPI involvement do you have in commissioning in this area? (d) What PPI involvement do you have in strategy development in this area? (e) What PPI involvement do you have in system development in this area? (f) What PPI involvement do you have in leadership and decision making in this area? (g) What PPI involvement do you have in service delivery in this area? (h) What changes have resulted from PPI involvement?

Outcomes and impact for older people and carers

(a) How do you measure outcomes for older people and carers? (b) What service changes have most affected older people and carers in this area? – What has been the impact?

(c) What has been the impact of particular initiatives to reduce unplanned admissions? – Refer to initiatives already mentioned above. – What is the patient experience of these services?

(d) Have outcomes for older people and carers changed over the last 5 years? – For the better? – For the worse?

(e) What changes are needed to improve emergency services and outcomes for older people 85+ and carers? (f) What are the benefits to older people and their carers of reducing unplanned admissions? (g) What are the disadvantages to older people and their carers of reducing unplanned admissions? (h) Have you carried out any patient satisfaction surveys in emergency/unplanned/urgent care in the last 5 years? – Can we have the report?

(i) Have you carried out any service evaluation looking at urgent care services for people over 85?
