# Supplementary material for: Understanding variation in unplanned admissions of people aged 85 and over: a systems-based approach
Source: BMJ Open. 2019 Jul 9;9(7):e026405. doi: 10.1136/bmjopen-2018-026405 (PMC6615796; doi:10.1136/bmjopen-2018-026405)
Supplement: Author's manuscript [file bmjopen-2018-026405.draft_revisions.pdf]

BMJ Open is committed to open peer review. As part of this commitment we make the peer review history of every article we publish publicly available.

When an article is published we post the peer reviewers' comments and the authors' responses online. We also post the versions of the paper that were used during peer review. These are the versions that the peer review comments apply to.

The versions of the paper that follow are the versions that were submitted during the peer review process. They are not the versions of record or the final published versions. They should not be cited or distributed as the published version of this manuscript.

BMJ Open is an open access journal and the full, final, typeset and author-corrected version of record of the manuscript is available on our site with no access controls, subscription charges or pay-per-view fees (http://bmjopen.bmj.com).

If you have any questions on BMJ Open's open peer review process please email info.bmjopen@bmj.com

BMJ Open

Understanding variation in acute admissions of people aged 85 and over: a systems-based approach

Journal:	*BMJ Open*
Manuscript ID	bmjopen-2018-026405
Article Type:	Research
Date Submitted by the Author:	01-Sep-2018

Complete List of Authors:	Wilson, Andrew; University of Leicester, Health Sciences<br>Baker, Richard; University of Leicester, Health Sciences<br>Bankart, John; University of Leicester, Health Sciences<br>Banerjee, J; University Hospitals of Leicester NHS Trust, Infirmary Square, Leiceste<br>Bhamra, Ran; Loughborough University<br>Conroy, Simon; University of Leicester<br>Kurtev, Stoyan; University of Leicester, Health Sciences<br>Phelps, Kay; University of Leicester College of Medicine Biological Sciences and Psychology, Health Sciences<br>Regen, Emma; University of Leicester College of Medicine Biological Sciences and Psychology, Health Sciences<br>Rogers, Stephen; University of Leicester, Department of Health Sciences<br>Waring, Justin; University of Nottingham, Centre for Health Innovation, Leadership & Learning / Nottingham University Business School

Keywords:	Older people, Unplanned admissions, Health systems, Mixed methods

SCHOLARONE™
Manuscripts

Understanding variation in acute admissions of people aged 85 and over: a systems based approach

Andrew Wilson (1), Richard Baker (1), John Bankart (1), Jay Banerjee (2), Ran Bhamra (3), Simon Conroy (1), Stoyan Kurtev (1), Kay Phelps (1), Emma Regen (1), Stephen Rogers (4) and Justin Waring (5)

(1) Department of Health Sciences, University of Leicester, Leicester, UK

(2) University Hospitals of Leicester NHS Trust, Leicester, UK

(3) Wolfson School of Mechanical and Manufacturing Engineering, Loughborough University, Loughborough, UK

(4) Department of Public Health, NHS Northamptonshire, Northampton, UK

(5) Business School, University of Nottingham, Nottingham, UK

Corresponding author

Professor Andrew Wilson
Department of Health Sciences
College of Life Sciences
University of Leicester, George Davies Centre
Leicester LE1 7RH

aw7@le.ac.uk

For peer review only

Understanding variation in acute admissions of people aged 85 and over: a systems-based approach

Abstract

Aim: To examine system characteristics associated with variations in unplanned admission rates in those aged 85+.

Design: Mixed methods

Setting: Primary Care Trusts in England were ranked according to changes in admission rates for people aged 85+ between 2007 and 2009, and study sites selected from each end of the distribution: three 'improving' sites where rates had declined by more than 4% and three 'deteriorating' sites where rates had increased by more than 20%. Each site comprised an acute hospital trust, its linked primary care trust/clinical commissioning group, the provider of community health services, and adult social care.

Participants: A total of 142 representatives from these organisations were interviewed to understand how policies had been developed and implemented. McKinsey's 7S framework was used as a structure for investigation and analysis.

Results: Although there were differences within the two groups of sites, improving sites reported more evidence of comprehensive system-wide strategies and strong leadership, enabling the development and implementation of policies and procedures for older people, shared across a more integrated health and social care setting. In these sites, primary and intermediate care services appeared more comprehensive and better integrated with other parts of the system, and policies in Emergency Departments were more focused on providing alternatives to admission.

Conclusions: Health and social care communities which have attenuated admissions of people aged 85+ prioritised developing a shared vision and strategy, with sustained implementation of a suite of interventions.

For peer review only

Strengths and limitations of this study'

- In England, unplanned hospital admissions for the people aged 85 and over are rising but there is substantial geographical variation
- A 'whole system approach' can be used to understand this variation between health economies
- Through qualitative interviews with a comprehensive range of informants, we examined three sites where rates of admission in this age group rose most sharply and three in which the rise was reversed or attenuated
- The study relied on institutional memory at a time of transition

For peer review only

Key words

Older people

Unplanned admissions

Health systems

Mixed methods

Understanding variation in acute admissions of people aged 85 and over: a systems-based approach

Word count: 3998

Introduction

In England between 2001/2 and 2012/13, unplanned admissions of people aged 65+ rose by 46% and the age-standardised rate by 25%. Rates of increase rose steadily with age: from 9.9% for those aged 65-69 to 50.2% for those aged 90+.(1)

Research consistently finds unexplained variations in the rise of unplanned admissions, suggesting lessons may be learned from different experiences.(2-5) Alternatives to admission are particularly important in those aged 85+, who often present with multiple comorbidities, polypharmacy, cognitive impairment and disability. Once admitted, they have longer stays, are more prone to hospital acquired complications, and may experience more difficulty returning to their usual place of residence.(6, 7) Furthermore, minimising time spent in hospital is a health outcome that matters to older people.(8) There is increasing evidence that alternatives to acute admission, such as Hospital at Home, produce similar if not better outcomes (9), although In England, provision of these services remains about half what is needed.(10)

Several initiatives have been introduced to address the increase in acute admissions.(2) There is good evidence that higher continuity in primary care is associated with fewer admissions(11), and that senior review in Emergency Departments (ED) can be effective in reducing admissions.(12) There is also some evidence of benefit from integrating primary and secondary care and health and social care.(13) However as the Kings Fund report concluded, 'a combination of interventions intended to reduce admissions may be expected to have a 'cumulative' effect'.(2) To understand variations in admissions it is therefore useful to examine the configuration of the whole system.

As most of the variation in admission rates is due to relatively immutable demographic factors(2), examining differing trends in rates over time provides an opportunity to explore the impact of changes in service configurations.

In this study, we aimed to identify system characteristics associated with higher and lower increases in unplanned admission rates in those aged 85+. This article comprises the qualitative component of a mixed method study.(14)

Methods

Methodology

We used a qualitative, multiple, explanatory case study approach as our principal method.(15) Following consideration of tools to enable the analysis of complex organisations, the McKinsey 7S framework was selected. [20] Its particular contribution was to help understand complexity (the systems based approach), and emphasise that for change to be effective, changes in any one component should be accompanied by complementary changes in others.

For peer review only

Site selection

Six study sites were selected based on admission data for patients aged 85+ from English primary care trusts (PCTs). These organisations were responsible for commissioning primary, community and secondary health services, and were succeeded by Clinical Commissioning Groups (CCGs) in 2013. We used PCTs as the basis of site selection as these had a population base to derive admission rates.

Admission rates for people aged 85+ were calculated from HES data (2007/08-2009/10).(16) Data were not available for some PCTs due to mergers. For the 143/154 (93%) PCTs for which we had data, a regression co-efficient was calculated for the change in admission rates over the three-year period, adjusting for population size and age, which was used to rank PCTs.

We selected three sites where rates of admission had increased most rapidly, and three where they had declined. Each site comprised an acute hospital trust, its linked PCT, the provider of community health services, and adult social care. An inclusion criterion was that more than 80% of acute admissions for people aged 85+ from the PCT were admitted to one acute Trust, so that there was at least a potential partnership between these organisations. Table 1 shows the ranking and admission rates for the selected sites: improving sites had an annual decrease in admission rates for those aged 85+ of 1-2%, and deteriorating sites an annual increase of 6%.

Quantitative data collection

After the sites had been selected we used updated HES data to examine 85+ admission rates over five years (2007/08 to 2011/12) rather than just the three years used for selection. We used the NHS portal (now NHS digital(17)) to examine admissions for acute and chronic ambulatory care sensitive conditions (ACSCs)(18) from 2007/08 to 2009/10. These are age adjusted rates per 100,000; data are not available for specific age groups.

Qualitative data collection

In each selected site, a key individual was identified in each organisation who advised on potential key informants. Further participants were identified by snowball sampling.(19) In 2013, two rounds of data collection were conducted. In the first round, an understanding of the system's history and drivers was sought in interviews with key high-level informants, including commissioners and managers of health and social care with responsibility for those aged 85+, and clinicians and care providers with leadership roles in primary care, emergency departments (ED), social care, intermediate and secondary care. In the second round, we examined specific components of the system, using in depth interviews and focus groups with those involved in delivering care, to explore issues involved in translating policy directives to changes in provision of care. These included clinicians in emergency departments and Acute Medical Units (AMUs), managers of intermediate and integrated care provision and clinicians in primary care. In each site, a focus group was convened including representatives of carers and service users to capture their perspectives.

Topic guides were based on the literature and agreed by the research team. For each site, data on population characteristics and admission rates were prepared to prompt discussion. Interviews were conducted at the workplace by research fellows (ER and KP) with substantial experience of policy focused qualitative work, who also made extensive fields notes for each site. Interviews and focus groups lasted 30-60 minutes and were recorded and transcribed verbatim.

For peer review only - http://bmjopen.bmj.com/site/about/guidelines.xhtml

Analysis

Qualitative data analysis was undertaken in a stepwise, interpretative approach. Firstly, all data from each case site was assigned to individual members of the project team for initial inductive, open coding. The second and main stage of data analysis involved two independent researchers developing detailed case reports. Following a framework approach (20) all data items were systematically scrutinised with data coded according to the 7S categories. NVivo software (21) was used to provide an audit trail. Guidance for coding was agreed by team members, including how items would be categorised according to the 7S framework. In line with the principle of constant comparison, each category was systematically checked for its internal consistency and inter-relationships.(22)

Patient and Public Involvement

The study was presented to the Leicester older people's research PPI forum at the planning stage, and a representative from this forum was a member of the Steering Group.

NHS ethical approval was not required as patients were not being interviewed. Ethics approval was obtained from University xx and R&D approvals from participating organisations. Informed consent was obtained from all participants.

Results

Quantitative findings

Table 2 presents data on rates of 85+ admissions between 2007/08 and 2011/12. In improving sites these increased by 3.3% (range -2.4% to 10.6%) and in deteriorating sites by 22.7% (range 16.3% to 29.2%). During the 5 year period, the mean linear regression slope per annum was 0.003 (0.3%) for improving sites and 0.022 (2.2%) for deteriorating sites. In general, rates of admission remained fairly stable in the additional two years we examined after site selection. Between using HES data for selection and this analysis, some corrections had been made to 2007/8 data.

Changes in rates of admission for ambulatory care sensitive conditions are also shown in table 2. In the three years examined, rates for acute conditions fell by 8.5% in improving sites (range -17.5% to 8.3%) and increased by 18.0% in deteriorating sites (range 4.8% to 26.8%). Mean rates of change differed less for chronic conditions, but with bigger variations between sites. They reduced by 3.9% in improving sites (range -7.4% to 0.4%) and increased by 1.0% in deteriorating sites (range -14.1% to 16.0%).

Qualitative findings

In total 142 informants contributed to either individual interviews or focus groups, as shown in table 3. The number of contributions from each site varied, in part because some agencies declined to participate. In site I2, community services and social services were provided by a single organisation.

Findings are presented by 7S themes. Quotes are identified by site and employing organisation of respondent (PCT/CCG: A; community services: B; acute trusts: C) and respondent number.

For peer review only

1. Strategy

This was defined as the plan of activity for the whole system, and alignment of the system to its goals. Improving sites exhibited more of a shared and comprehensive system-wide strategy for managing unplanned care, including specific policies and procedures for older people. These strategies and policies were shared across the wider health and social care system suggesting an underlying basis of collaboration and coordination, and a reduced risk of system dominance by one provider.

> *We had an audacious programme goal, which was all about reducing emergency admissions, and we had quite a lot of buy-in … to a whole-system approach. I1 A-01*

> *For the whole time I've been here, it (urgent care) has been a top priority. We want urgent care pathways (including ambulance, ED and admissions) to be as high quality as possible. I3-A-02*

Deteriorating sites revealed less evidence of a system strategy. Although individual system components had developed strategies for aspects of unplanned care, such as reducing length of stay, there was less appreciation of how the components of the wider health system should work together. Strategies tended to be dominated by acute care provision to the detriment of policies to expand primary and community care.

> *The system plans have bullets like 'we'll support care close to home but we'll support financial sustainability of the acute hospitals.' Unless you have some sort of integration, those two things are mutually exclusive in the long term. D1-A-03*

An important difference between improving and deteriorating sites was their approach to improvement projects. In improving sites, these were generally well-resourced, often through funding arrangements linked to national initiatives. Moreover, they were usually given time to develop and embed into practice, rather than being subject to changing fashions or emerging policies. In contrast, in deteriorating sites projects tended to be more reactive and short-lived with little follow through.

> *We've piloted lots of good things, but it's been the usual story of just doing pilots and not doing them at sufficient scale, we've dabbled in things and haven't really followed them sufficiently through. D3-A-03*

2. Structure

This was defined as how different components of the system related to each other. In improving sites there was closer integration of primary, acute and community services. This was facilitated by fewer organisations providing services and clearer geographical boundaries.

> *X was one of the few dedicated Community Trusts in the country...there's not several different providers. Patients can't get moved around the system because there's only basically GP practices, intermediate tier and one acute trust.I1-B-01*

> *The care trust, .. was integrated – the health organisation held the social care budget on behalf of the council .. that alone meant that the approach to commissioning was truly integrated at the budget and organisational level. So that I think is fundamental. I2-A-01*

> *We hardly have any cross-boundary issues, it's just one social services department, one acute trust, one community trust, one mental health trust and one health commissioner – that's it. I3-A-02*

In deteriorating sites, there was less evidence of integration between acute, primary and community services. Governance and funding arrangements were more complex, with different ways of working. There was often emphasis on key care stages, such as admission and discharge, but not on the wider constellation of agencies, handovers and transitions that patients face across the system.

> *The services are so complex that we as health professionals find it difficult and I think … for somebody who is over 85 or for anybody I think - it's really difficult for them to understand where to go for help and I think so by default people know that this (A and E) is open 24 hours a day, you can literally just walk in and you'll be helped. D1-C-01*

> *I don't think it [unplanned admissions], is managed very well really in this area which is one of the reasons why you see relatively high admission rates. Part of the reason for that is because there are separate organisations each with their own agendas and each with their own pressures.D2-C-01*

3. Systems

This was defined as individual services contributing to the whole system. The most consistent differences between improving and deteriorating sites were in GP services, intermediate care and ED provision.

General practice

In improving sites, general practice appeared better supported financially, more innovative, for example with IT systems, and better integrated with other providers. There was closer alignment of out-of-hours GP services with either community or acute NHS providers, which facilitated closer integration of primary, acute and community services, especially for information sharing and continuity of care.

> *Round here I think GPs are more proactive in managing their patients. If you're a GMS [General Medical Services] practice there's no incentive for you keeping your admission. We had our PBC [practice-based commissioning] budgets and if we were within our budget there'd be some financial reward for that. I2-A-01*

> *(IT system) facilitates three-way conversations; the GP will be on the line, the nurse would be on the line and the acute physician from AMU [the acute medical unit] would be on the line and having a conference about whether or not it's appropriate that person ought to go to hospital. I1-A-01*

In deteriorating sites there was a sense of under-investment and insufficient planning for primary care. GP practices were seen as providing a more limited set of services with problems of access due

in part to single handed practices and half day closures, thereby increasing demand on Emergency Departments (EDs). Changes in out of hours GP provision was also thought to impact on ED attendances.

> *There's been underinvestment in primary care, there hasn't been a clear primary care strategy… there's been underinvestment in primary care local enhanced services compared to other places. D3-A-03*

> *We've got a high proportion of (older) GPs, it's I think over 50% who have retired, taken their pension and come back. So there is no motivation for them to change at all. D1-A-02*

Intermediate Care

Intermediate care provision at scale was widely seen to be important. This was more developed at improving sites but even here, short term funding meant services were not stable. Key elements of provision included a single point of access, a unified system often from a single provider, and multidisciplinary working to reduce duplication.

> *We have a single point of access manned by advice officers, behind which there are three levels of triage– at every level of triage it's an integrated triage between health and social care.I2-A-03*

> *The multidisciplinary teams in intermediate care have made a difference– you know, sometimes, one person's seeing a physio, an OT and a nurse, and they just need to see one member of the team. I1- B-02*

Emergency Departments

There was agreement that skill mix in ED could influence the number of older patients admitted. Initiatives in improving sites included provision of geriatricians in ED, more senior staffing and involvement of GPs. Linked to this were initiatives to provide GP with accessible alternatives to acute admission, such as rapid access clinics or telephone consultations with a geriatrician.

> *GPs in A & E – well GPs have been in the hospital ever since I've been here, so they've either been co-located, so very close to A & E or in A & E. I2-A-02*

> *We introduced our (older people's) clinic. If they (GPs) have got an elderly patient with them in the surgery, they're just not sure what to do, they just pick up this phone line, and there's a geriatrician who will advise them. I3-C-04*

4. Shared values, style, staff and skills

These components are presented together as there was overlap between values and style. Additionally, some issues relating to staffing and skills have been covered in earlier sections as these seemed more relevant to specific components rather than the system as a whole (for example upskilling staff in ED). Shared values within and between components of the system appeared particularly important. Improving sites were characterised by stable staffing and leadership that

For peer review only

supported continuity of purpose, fostered trust and collaborative working, and maintained commitment to improvement. In contrast, deteriorating sites had higher staff turnover and appeared more distracted by short term changes in policy.

> *It (stability) helps in terms of building those relationships and building trust and allowing us to perhaps take more risks. I2-A-02*

> *I think the things that work really well are the relationships at an operational, and to a degree going into strategic work, and I think those individual relationships, and people knowing each other and having a level of trust. I3-C-02*

> *No-one's in place long enough … the natural political cycle is shorter than the natural planning cycle for the health system. D3-A-01*

Several other values emerged from the interviews. These included a general belief that admission can be counterproductive for older people and the need to challenge a mind-set that admission is the default option.

> *Acute hospitals are fundamentally not the right place for over eighty fives..,. you're far better in your home environment or in a supported environment that's not hospital..D3-C-01*

> *Well, there is a culture of admission in this hospital –someone comes to the front door, they see a junior doctor, admit them. I mean, you know, if I go on a post-take ward round and see a patient that's been admitted to one of these wards on the same day and say they can be discharged, the nurse looks at me as though I'm some sort of idiot: 'What are you talking about discharge? I haven't finished admitting them yet. D1-C-02*

Several sites described a clash of values between commissioners, managers and clinicians, particularly when manging older people, and the importance of clinical leadership.

> *No, there's nobody clinical, there's nobody caring, there's nobody who actually does the business of looking after people, particularly the frail elderly, who are messy and don't fit into a clear protocol.I2-A-05*

> *I think what we probably need now is clinically led provider organisations cause you've still got managerial led provider organisations. The doctors do talk to each other but then they don't talk to the managers and the managers talk about a different thing so it's not necessarily connected. D1-A-02*

Discussion

Summary of findings

We found some important differences between sites in which admission rates for people aged 85+ had increased most rapidly and sites in which these rates had stabilised or declined. In improving sites there was more evidence of strong strategic leadership, enabling the development of a comprehensive system-wide strategy, including specific policies and procedures for older people, which were shared across a more integrated health and social care setting. This encouraged longer-term, consistent development of strategies, often in the face of changing national imperatives. This

For peer review only

stability also allowed trust and shared commitment to be established and the emergence of common values across the system.

In improving sites, primary care appeared stronger, both in terms of service provision and strategic engagement. This could be one reason why admissions for ambulatory care sensitive conditions fell on average in improving sites and increased in deteriorating sites. There is also evidence presented in our funder's report (14) that GP access was better in improving sites. Intermediate care was also more developed in improving sites. These services appeared to work best when provided at scale and fully integrated with each other, offering round-the-clock availability with a single point of access, shared information systems and specialist nursing and geriatric support. Improving sites also seemed more equipped to reduce admissions of older people from ED, through a variety of initiatives, including more senior staffing, involvement of GPs and provision of specialist nurses or geriatricians. In summary, improving sites generally made fuller use of a suite of strategies to reduce unplanned admissions, and importantly had a more system-wide outlook and strategic approach.

Comparison with previous studies

Findings from several recent qualitative and mixed methods studies are consistent with those reported here. A study examining six emergency and urgent care sites suggested that improving GP out of hours access, senior review in ED and multidisciplinary teams could reduce admissions(23), and others have emphasised how the culture and staffing in ED can influence admission rates (24, 25). We are not aware of previous qualitative work examining the whole system, but our findings support recommendations on integrated care made by the Kings Fund, including 'sharing sovereignty', developing a persuasive vision and establishing leadership.(26). More recently a CQC report concluded: 'To truly coordinate care, local system leaders must ensure there is a golden thread linking vision to delivery, so that everyone involved can not only share the vision but see themselves as part of the team that delivers it'.(27)

Strengths and limitations

Our study design offered strong internal validity through in-depth analysis within case and structured comparison between cases.(28) The design and conduct of this study reflects a number of features to enhance trustworthiness.(29). The approach allowed a common method of data collection and analysis subject to open scrutiny by the wider research team and advisors, enhancing dependability. In terms of confirmability, the researchers were supported in being reflexive about their own role in data collection, including review meetings with the wider research team.

That researchers and participants knew how each site had performed helped to inform the interviews; by presenting data on admissions we were able to engage in a more detailed discussion of issues and strategies. It is unlikely that informants would be unaware of their own performance. However this approach may have meant that both interviewers and participants may have focused on what was perceived to be working well in improving sites, and on more negative issues in deteriorating sites.

For peer review only

The study had several other limitations. Our selection criteria were based on changes in historic data; the additional data on admissions for 2010/11 and 2011 showed that over the five year period differences between improving and deteriorating sites persisted but in most sites stabilised somewhat over the last two years, and the trajectory of improvement or deterioration slowed. Furthermore there were differences in performance within improving and deteriorating groups. Some informants found it challenging to reflect on past events rather than the current situation. The strongest interview data came from informants who had been in post for the period of interest and so able to provide an institutional memory. This problem was compounded by the fact the study was conducted during a period of organisational upheaval in the NHS, leading to many informants being relatively new in post.

The pace of change within the NHS has quickened since the study was conducted, with additional challenges imposed by austerity. However, we feel our key messages remain relevant as they emphasise the need for strategies and stability that can weather the impact of ongoing changes. We also acknowledge that sites with a more complex mix of acute trusts (which we deliberately excluded) may experience different or additional problems and solutions.

The McKinsey 7S framework was effective in enabling the systematic investigation of system components and their interaction, but was less useful in mapping more abstract issues such as style and values. The framework appeared better suited to examining individual organisations, rather than large complex systems of inter-dependent heterogeneous system actors, which may have been further understood and analysed using, for example, Beer's Viable Systems Model (22).

Implications for policy makers and service providers

This study supports taking a whole system approach in designing services for older people. This is best achieved by developing a sustained and shared vision focusing on outcomes that matter to older people(8), establishing strong leadership without dominance by one organisation, maximising integration and minimising complexity within the system. These findings have potential to inform the practical implementation in England of Strategic Transformation Partnerships and Accountable Care Organisations, vehicles designed to promote system-based collaboration. (30)

Conclusions

Health and social care communities which attenuated unplanned admissions of people aged 85+ prioritised developing a shared vision and strategy, encompassing multiple organisations and backed by strong leadership and shared values. This allowed sustained implementation of a suite of interventions, including better-developed primary and intermediate care services working closely with the hospital and emergency departments.

For peer review only

Data sharing

Full transcripts are available from the corresponding author.

Contributions

All authors contributed to study design, and analysis, and have approved the final manuscript. Andrew Wilson led the study, Richard Baker and Steven Rogers contributed expertise in primary care and quality improvement, John Bankart and Stoyan Kurtev led on selection of sites. Jay Banerjee and Simon Conroy constructed expertise on ED policies and geriatric medicine respectively. Ran Bhamra contributed expertise on system theory and Justin Waring led the analysis.
Kay Phelps and Emma Regen led on fields work and conducted most of the interviews.

Acknowledgements

We are grateful for the support of members of the steering group throughout the project, and for advice from the Leicestershire, Northamptonshire and Rutland Older People's Research Patient and Public Involvement Forum. Access to sites was facilitated by the NIHR Clinical Research network. Most of all we would like to acknowledge participants at all study sites for their time and the candid insights they provided.

Conflict of interest

The authors declared no potential conflicts of interest with respect to the research, authorship and/or publication of this article.

Funding

The project was funded by the NIHR Health Service and Delivery and Research Programme (project number 10/1010/05). The views and opinions expressed in this article are those of the authors and do not necessarily reflect those of the Health Service and Delivery and Research Programme, NIHR, NHS or the Department of Health.

For peer review only

References

1. Raphael Wittenberg LS, Barry McCormick, Jeremy Hurst. Understanding emergency hospital admissions of older people. Oxford: 2014.
2. Purdy S. Avoiding hospital admissions - What does the research evidence say? London: Kings Fund, 2010.
3. University of Birmingham Health Services Management Centre. Reducing Unplanned Hospital Admissions: what does the literature tell us? Birmingham: 2006.
4. Tian Y, Dixon A, Gao H. Emergency hospital admissions for ambulatory care sensitive condtions: identifying the potential for reducations. London: The Kings Fund, 2012.
5. O'Cathain A, Knowles E, Maheswaran R, Pearson T, Turner J, Hirst E, et al. A system-wide approach to explaining variation in potentially avoidable emergency admissions: national ecological study. BMJ Quality & Safety. 2014;23(1):47-55.
6. Sager MA, Franke T, Inouye SK, Landefeld CS, Morgan TM, Rudberg MA, et al. Functional Outcomes of Acute Medical Illness and Hospitalization in Older Persons. Arch Intern Med. 1996;156(6):645-52.
7. Landeiro F, Roberts K, Gray AM, Leal J. Delayed Hospital Discharges of Older Patients: A Systematic Review on Prevalence and Costs. The Gerontologist. 2017:gnx028.
8. Akpan A, Roberts C, Bandeen-Roche K, Batty B, Bausewein C, Bell D, et al. Standard set of health outcome measures for older persons. BMC Geriatrics. 2018;18(1):36.
9. Shepperd S, Iliffe S, Doll H, Clarke M, Kalra L, Wilson AD, et al. Admission avoidance hospital at home. Cochrane Database of Systematic Reviews [Internet]. 2016; (9). Available from: http://onlinelibrary.wiley.com/doi/10.1002/14651858.CD007491.pub2/abstract;jsessionid=D3133E6EEEA6E31B6A45F33C66800223.f03t02
10. NHS Benchmarking Network. National Audit of Intermediate Care. Manchester: NHS Benchmarking Network, 2013.
11. Barker I, Steventon A, Deeny SR. Association between continuity of care in general practice and hospital admissions for ambulatory care sensitive conditions: cross sectional study of routinely collected, person level data. BMJ. 2017;356.
12. White AL, Armstrong PAR, Thakore S. Impact of senior clinical review on patient disposition from the emergency department. Emergency Medicine Journal. 2010;27(4):262-5.
13. Damery S, Flanagan S, Combes G. Does integrated care reduce hospital activity for patients with chronic diseases? An umbrella review of systematic reviews. BMJ Open. 2016;6(11).
14. Wilson A, Baker R, Bankart J, Banerjee J, Bhamra R, Conroy S, et al. Establishing and implementing best practice to reduce unplanned admissions in those 85 years and over through system change (Establishing System Change for Admissions of People 85+(ESCAPE 85+)): a mixed-methods case study approach. Health Services and Delivery Research. 2015;3:37.
15. Yin RK. Multiple case studies with embedded sub cases. Case Study Research: Design and Methods. Fourth Edition ed. California: Sage Publications; 2009.
16. NHS_Digital. Hospital Episode Statistics.
17. NHS_digital. Emergency hospital admissions: acute conditions usually managed in primary care: indirectly standardised rate, all ages, annual trend, F,M,P 2016 [cited 15/05/2018]. Available from: https://digital.nhs.uk/data-and-information/publications/clinical-indicators/compendium-of-population-health-indicators/compendium-hospital-care/current/emergency-admissions/emergency-hospital-admissions-acute-conditions-usually-managed-in-primary-care-indirectly-standardised-rate-all-ages-annual-trend-f-m-p.
18. Purdy S, Griffin T, Salisbury C, Sharp D. Ambulatory care sensitive conditions: terminology and disease coding need to be more specific to aid policy makers and clinicians. Public Health. 2009;123(2):169-73.
19. Marshall MN. The key informant technique. Family Practice. 1996;13(1):92-7.

For peer review only - http://bmjopen.bmj.com/site/about/guidelines.xhtml

20. Gale N, Heath G, Cameron E, Rashid S, Redwood S. Using the framework method for the analysis of qualitative data in multi-disciplinary health research. BMC Medical Research Methodology. 2013;13(1):117.
21. NVivo qualitative data analysis software. QSR International Pty Ltd Version 7. 2006.
22. Boeije H. A purposeful approach to the constant comparative method in the analysis of qualitative interviews. Qual Quant. 2002;36(4):391 - 409.
23. O'Cathain A, Knowles E, Turner J, Hirst E, Goodacre S, Nicholl J. Variation in avoidable emergency admissions: multiple case studies of emergency and urgent care systems. Journal of Health Services Research & Policy. 2016;21(1):5-14.
24. Pinkney J RS, Benger J, Brant H, Joel-Edgar S, Swancutt D, et al. How can frontline expertise and new models of care best contribute to safely reducing avoidable acute admissions?

A mixed-methods study of four acute hospitals. Health Serv Deliv Res. 2016;4(3).
25. Pope I, Burn H, Ismail SA, Harris T, McCoy D. A qualitative study exploring the factors influencing admission to hospital from the emergency department. BMJ Open. 2017;7(8).
26. Ham C, Walsh N. Making integrated care happen at scale and pace. London: The Kings Fund; 2013.
27. Commission CQ. The state of health care and adult social care in England 2016/17. London: Care Quality Commission 2017.
28. Mintzberg H, Lampel JB, Quinn J, Ghoshal S. The Strategy Process: Concepts, Contexts, Cases, 5/E. Harlow: Pearson; 2014.
29. Lincoln YS, Guba EG. Establishing trustworthiness. London: Sage; 1985. 289-331 p.
30. NHS_Providers. STPs and accountable care: background briefing. 2018.

For peer review only

PCT<br>Rank for slope (n=143)	85+ admission rate (number of admissions/100 population aged 85+)			Slope (per annum change)	% change in rate	% admissions to linked hospital trust	% aged 85+	Reference in paper
	2007/08	2008/09	2009/10					
4	0.55	0.51	0.51	-0.02	-7.3	89	2.6	I1
5	0.61	0.6	0.57	-0.02	-6.6	87	2.6	I3
9	0.41	0.41	0.39	-0.01	-4.9	83	2.2	I2
132	0.48	0.54	0.59	0.06	22.9	92	2.2	D1
133	0.41	0.45	0.52	0.06	26.8	87	1.7	D3
135	0.49	0.59	0.61	0.06	25.5	83	1.8	D2

Table 1: Selection of improving (I) and deteriorating (D) Primary Care Trusts (PCT)

For peer review only

	I1	I2	I3	D1	D2	D3		Improving sites average *	Deteriorating sites average *
**85+ admissions/100 aged 85+ per annum**									
2007/08	0.47	0.41	0.57	0.48	0.49	0.40		0.48	0.46
2008/09	0.51	0.42	0.6	0.55	0.59	0.45		0.51	0.53
2009/10	0.51	0.40	0.58	0.60	0.61	0.53		0.50	0.58
2010/11	0.54	0.39	0.58	0.60	0.56	0.48		0.50	0.55
2011/12	0.52	0.40	0.58	0.62	0.57	0.49		0.50	0.56
% change between 2007/8 and 2011/12	10.64	-2.44	1.75	29.17	16.33	22.50		3.32	22.67
Linear regression slope (per annum change in rate)	0.013	-0.005	0	0.033	0.013	0.020		0.003	0.022
**Acute ACSC all ages. Indirectly age and sex standardised rate per 100,000**									
2007/08	424	579	462	367	397	619		488	461
2008/09	515	591	347	388	461	699		484	516
2009/10	350	627	387	449	416	785		455	550
% change between 2007/8 and 2009/10	-17.45	8.29	-16.23	22.34	4.79	26.82		-8.46	17.98
Linear regression slope (per annum change in rate)	-37	24	-37.5	41	9.5	83		-16.83	44.50
**Chronic ACSC all ages. Indirectly age and sex standardised rate per 100,000**									
07/08	220	240	148	188	177	247		203	204
08/09	276	247	104	212	177	265		209	218
09/10	210	241	137	218	152	249		196	206
% change between 2007/8 and 2009/10	-4.55	0.42	-7.43	15.96	-14.12	0.81		-3.85	0.88
Linear regression slope (per annum change in rate)	-5	0.5	-5.5	15	-12.5	1		-3.33	1.17

Table 2: Changes in admission rates for patients aged 85+, and rates of admission for acute and chronic ambulatory care sensitive conditions (ACSC) in improving and deteriorating sites

*Averages derived from rows not columns.

For peer review only

For peer review only - http://bmjopen.bmj.com/site/about/guidelines.xhtml

Site	Acute Trust	PCT/CCG	Community Services	Social services	PPI	Total
I1	declined	2	13 (7 individual, 2 focus groups [n=2 and 4])	declined		15
I2	6	6	16 (7 individual, 2 focus groups [n=9])		Focus group (n= 5)	33
I3	5	3	24 (4 individual, 3 focus groups [n=6,6,8])	2	Focus group (n=9)	43
D1	7	3	3	2	Focus group (n=5)	20
D2	10	3	2	4	Focus group (n=5)	24
D3	2	3	declined	1	1	7
Total	30	20	58	9	25	142

Table 3. Informants by site and organisational category

For peer review only

For peer review only

Consolidated criteria for reporting qualitative studies (COREQ): 32-item checklist

Developed from:
Tong A, Sainsbury P, Craig J. Consolidated criteria for reporting qualitative research (COREQ): a 32-item checklist for interviews and focus groups. *International Journal for Quality in Health Care*. 2007. Volume 19, Number 6: pp. 349 – 357

No. Item	Guide questions/description	Reported on Page #
**Domain 1: Research team and reflexivity**		
Personal Characteristics		
1. Inter viewer/facilitator	Which author/s conducted the inter view or focus group?	4
2. Credentials	What were the researcher's credentials? E.g. PhD, MD	4
3. Occupation	What was their occupation at the time of the study?	4
4. Gender	Was the researcher male or female?	Author list
5. Experience and training	What experience or training did the researcher have?	4
Relationship with participants		
6. Relationship established	Was a relationship established prior to study commencement?	no
7. Participant knowledge of the interviewer	What did the participants know about the researcher? e.g. personal goals, reasons for doing the research	no
8. Interviewer characteristics	What characteristics were reported about the inter viewer/facilitator? e.g. Bias, assumptions, reasons and interests in the research topic	no

For peer review only

**Domain 2: study design**		
Theoretical framework		
9. Methodological orientation and Theory	What methodological orientation was stated to underpin the study? e.g. grounded theory, discourse analysis, ethnography, phenomenology, content analysis	3
Participant selection		
10. Sampling	How were participants selected? e.g. purposive, convenience, consecutive, snowball	3
11. Method of approach	How were participants approached? e.g. face-to-face, telephone, mail, email	3
12. Sample size	How many participants were in the study?	4
13. Non-participation	How many people refused to participate or dropped out? Reasons?	4
Setting		
14. Setting of data collection	Where was the data collected? e.g. home, clinic, workplace	4
15. Presence of non-participants	Was anyone else present besides the participants and researchers?	no
16. Description of sample	What are the important characteristics of the sample? e.g. demographic data, date	Table 2
Data collection		
17. Interview guide	Were questions, prompts, guides provided by the authors? Was it pilot tested?	4
18. Repeat interviews	Were repeat inter views carried out? If yes, how many?	no
19. Audio/visual recording	Did the research use audio or visual recording to collect the data?	4
20. Field notes	Were field notes made during and/or after the inter view or focus group?	4
21. Duration	What was the duration of the inter views or focus group?	4
22. Data saturation	Was data saturation discussed?	
23. Transcripts returned	Were transcripts returned to participants for comment and/or correction?	no
**Domain 3: analysis and findings**		
Data analysis		

For peer review only

24. Number of data coders	How many data coders coded the data?	4
25. Description of the coding tree	Did authors provide a description of the coding tree?	4
26. Derivation of themes	Were themes identified in advance or derived from the data?	4
27. Software	What software, if applicable, was used to manage the data?	4
28. Participant checking	Did participants provide feedback on the findings?	no
Reporting		
29. Quotations presented	Were participant quotations presented to illustrate the themes/findings? Was each quotation identified? e.g. participant number	throughout
30. Data and findings consistent	Was there consistency between the data presented and the findings?	throughout
31. Clarity of major themes	Were major themes clearly presented in the findings?	throughout
32. Clarity of minor themes	Is there a description of diverse cases or discussion of minor themes?	Minor themes

For peer review only

BMJ Open

Understanding variation in acute admissions of people aged 85 and over: a systems-based approach

Journal:	*BMJ Open*
Manuscript ID	bmjopen-2018-026405.R1
Article Type:	Research
Date Submitted by the Author:	12-Feb-2019
Complete List of Authors:	Wilson, Andrew; University of Leicester, Health Sciences<br>Baker, Richard; University of Leicester, Health Sciences<br>Bankart, John; University of Leicester, Health Sciences<br>Banerjee, J; University Hospitals of Leicester NHS Trust, Infirmary Square, Leiceste<br>Bhamra, Ran; Loughborough University<br>Conroy, Simon; University of Leicester<br>Kurtev, Stoyan; University of Leicester, Health Sciences<br>Phelps, Kay; University of Leicester College of Medicine Biological Sciences and Psychology, Health Sciences<br>Regen, Emma; University of Leicester College of Medicine Biological Sciences and Psychology, Health Sciences<br>Rogers, Stephen; University of Leicester, Department of Health Sciences<br>Waring, Justin; University of Nottingham, Centre for Health Innovation, Leadership & Learning / Nottingham University Business School
**Primary Subject Heading**:	Geriatric medicine
Secondary Subject Heading:	Health services research
Keywords:	Older people, Unplanned admissions, Health systems, Mixed methods

SCHOLARONE™
Manuscripts

Understanding variation in acute admissions of people aged 85 and over: a systems based approach

Andrew Wilson (1), Richard Baker (1), John Bankart (1), Jay Banerjee (2), Ran Bhamra (3), Simon Conroy (1), Stoyan Kurtev (1), Kay Phelps (1), Emma Regen (1), Stephen Rogers (4) and Justin Waring (5)

(1) Department of Health Sciences, University of Leicester, Leicester, UK

(2) University Hospitals of Leicester NHS Trust, Leicester, UK

(3) Wolfson School of Mechanical, Electrical and Manufacturing Engineering, Loughborough University, Loughborough, UK

(4) Department of Public Health, NHS Northamptonshire, Northampton, UK

(5) Business School, University of Nottingham, Nottingham, UK

Corresponding author

Professor Andrew Wilson
Department of Health Sciences
College of Life Sciences
University of Leicester, George Davies Centre
Leicester LE1 7RH

aw7@le.ac.uk

For peer review only

Understanding variation in acute admissions of people aged 85 and over: a systems-based approach

Abstract

Aim: To examine system characteristics associated with variations in unplanned admission rates in those aged 85+.

Design: Mixed methods

Setting: Primary Care Trusts in England were ranked according to changes in admission rates for people aged 85+ between 2007 and 2009, and study sites selected from each end of the distribution: three 'improving' sites where rates had declined by more than 4% and three 'deteriorating' sites where rates had increased by more than 20%. Each site comprised an acute hospital trust, its linked primary care trust/clinical commissioning group, the provider of community health services, and adult social care.

Participants: A total of 142 representatives from these organisations were interviewed to understand how policies had been developed and implemented. McKinsey's 7S framework was used as a structure for investigation and analysis.

Results: In general, improving sites provided more evidence of comprehensive system focused strategies backed by strong leadership, enabling the development and implementation of policies and procedures to avoid un-necessary admissions of older people. In these sites, primary and intermediate care services appeared more comprehensive and better integrated with other parts of the system, and policies in Emergency Departments were more focused on providing alternatives to admission.

Conclusions: Health and social care communities which have attenuated admissions of people aged 85+ prioritised developing a shared vision and strategy, with sustained implementation of a suite of interventions.

For peer review only

Strengths and limitations of this study'

- In England, unplanned hospital admissions for the people aged 85 and over are rising but there is substantial geographical variation.
- A 'whole system approach' can be used to understand this variation between health economies.
- Through qualitative interviews with a comprehensive range of informants, we examined three sites where rates of admission in this age group rose most sharply and three in which the rise was reversed or attenuated.
- The study relied on institutional memory at a time of transition.

For peer review only

Key words

Older people

Unplanned admissions

Health systems

Mixed methods

Understanding variation in acute admissions of people aged 85 and over: a systems-based approach

Word count: 4602

Introduction

Internationally, unplanned hospital admissions have increased steadily over recent decades.(1-4) In England between 2001/2 and 2012/13, unplanned admissions of people aged 65+ rose by 46% and the age-standardised rate by 25%. Rates of increase rose steadily with age: from 9.9% for those aged 65-69 to 50.2% for those aged 90+.(5)

Research consistently finds unexplained variations in the rise of unplanned admissions, suggesting lessons may be learned from different experiences.(6-9) Alternatives to admission are particularly important in those aged 85+, who often present with multiple comorbidities, polypharmacy, cognitive impairment and disability. Once admitted, they have longer stays, are more prone to hospital acquired complications, and may experience more difficulty returning to their usual place of residence.(10, 11) Furthermore, minimising time spent in hospital is a health outcome that matters to older people.(12) There is increasing evidence that alternatives to acute admission, such as Hospital at Home, produce similar if not better outcomes (13), although in England, provision of these services remains about half what is needed.(14)

Several initiatives have been introduced to address the increase in acute admissions.(6) There is good evidence that higher continuity in primary care is associated with fewer admissions(15), and that senior review in Emergency Departments (ED) can be effective in reducing admissions.(16) There is also some evidence of benefit from integrating primary and secondary care and health and social care.(17) However as the Kings Fund report concluded, 'a combination of interventions intended to reduce admissions may be expected to have a 'cumulative' effect'.(6) To understand variations in admissions it is therefore useful to examine the configuration of the whole system.

As most of the variation in admission rates is due to relatively immutable demographic factors(6), examining differing trends in rates over time provides an opportunity to explore the impact of changes in service configurations.

In this study, we aimed to identify system characteristics associated with higher and lower increases in unplanned admission rates in those aged 85+. This article focuses on the qualitative component of a mixed method study reported fully elsewhere(18)

Methods

Methodology

We used a qualitative, multiple, explanatory case study approach as our principal method.(19) Following consideration of tools to enable the analysis of complex organisations, the McKinsey 7S framework was selected as shown in Figure 1 [20] Its particular contribution was to help understand

For peer review only

the inherent complexity of the system as a whole, and emphasise that for change to be effective, changes in any one component should be accompanied by complementary changes in others.

Site selection

Six study sites were selected based on admission data for patients aged 85+ from English primary care trusts (PCTs). These organisations were responsible for commissioning primary, community and secondary health services, and were succeeded by Clinical Commissioning Groups (CCGs) in 2013. We used PCTs as the basis of site selection as these had a population base to derive admission rates.

Admission rates for people aged 85+ were calculated from HES data (2007/08-2009/10).(20) Data were not available for some PCTs due to mergers. For the 143/154 (93%) PCTs for which we had data, a regression co-efficient was calculated for the change in admission rates over the three-year period, adjusting for population size and age, which was used to rank PCTs.

After applying our inclusion and exclusion criteria (see below) we selected the three sites where rates of admission had increased most rapidly, and the three where they had declined most rapidly. Each site comprised an acute hospital trust, its linked PCT, the provider of community health services, and adult social care. An inclusion criterion was that more than 80% of acute admissions for people aged 85+ from the PCT were admitted to one acute Trust, so that there was at least a potential partnership between these organisations. Sites were excluded if they were known to be experiencing significant reconfiguration as reflected in publicly available information. Table 1 shows the ranking and admission rates for the selected sites: improving sites had an annual decrease in admission rates for those aged 85+ of 1-2%, and deteriorating sites an annual increase of 6%.

Quantitative data collection

After the sites had been selected we used updated HES data to examine 85+ admission rates over five years (2007/08 to 2011/12) rather than just the three years used for selection. We used the NHS portal (now NHS digital(21)) to examine admissions for acute and chronic ambulatory care sensitive conditions (ACSCs)(22) from 2007/08 to 2009/10. These are age adjusted rates per 100,000; data are not available for specific age groups. In the selected sites, invitations to participate were sent to the chief executives of the PCT and acute trust. In all cases, there was initial agreement from both parties. We then invited participation from the organisation responsible for community health services and social services.

Qualitative data collection

In each selected site, a key individual was identified in each organisation who advised on potential key informants. Further participants were identified by snowball sampling.(23) In 2013, two rounds of data collection were conducted. In the first round, an understanding of the system's history and drivers was sought in interviews with key high-level informants, including commissioners and managers of health and social care with responsibility for those aged 85+, and clinicians and care providers with leadership roles in primary care, emergency departments (ED), social care, intermediate and secondary care. In the second round, we examined specific components of the system, using in-depth interviews and focus groups with those involved in delivering care, to explore issues involved in translating policy directives to changes in provision of care. These included clinicians in Emergency Departments and Acute Medical Units (AMUs), managers of intermediate

For peer review only

and integrated care provision and clinicians in primary care. In each site, a focus group was convened including representatives of carers and service users to capture their perspectives.

Topic guides were based on the literature and agreed by the research team. The structure of the topic guide was as follows: views on unplanned admissions, views on system characteristics, specific questions on system characteristics, changes and recommendations, PPI involvement, outcomes. A detailed topic guide is presented in appendix 1.

For each site, data on population characteristics and admission rates were prepared to prompt discussion. Interviews were conducted at the workplace by research fellows (ER and KP) with substantial experience of policy focused qualitative work, who also made extensive fields notes for each site. Interviews and focus groups lasted 30-60 minutes and were recorded and transcribed verbatim.

Analysis

Qualitative data analysis was undertaken in a stepwise, interpretative approach. Firstly, all data from each case site was assigned to individual members of the project team for initial inductive, open coding. The second and main stage of data analysis involved two independent researchers developing detailed case reports. Following a framework approach (24) all data items were systematically scrutinised with data coded according to the 7S categories. These codes and descriptions provided the basis of regular discussion amongst the multi-disciplinary research team, first for reviewing the consistency of the analysis process (inter-coder), i.e. so that similar codes related to similar phenomena; and then for debating and agreeing the thematic interpretation of data. As part of this process the similarities and differences between codes were analysed, especially when relating data the 7S model, to ensure that data/codes were categorised in ways that were sufficiently distinct, or where they shared common or complementary features they were aggregated into higher order codes, which was especially important when relating the data to the McKinsey model. NVivo software (25) was used to provide an audit trail. Guidance for coding was agreed by the team , including how items would be categorised according to the 7S framework. In line with the principle of constant comparison, each category was systematically checked for its internal consistency and inter-relationships.(26) Through the processes of analysis and interpretation the research team was especially concerned to test the reliability and confidence of interpretation through looking for counterfactuals in the data that could represent contingencies to the emerging interpretations. Illustrative and exemplar extracts of data are provided in the subsequence results section, and further empirical data can be found in the main study report.

Patient and Public Involvement

The study was presented to the Leicester older people's research PPI forum at the planning stage, and a representative from this forum was a member of the Steering Group.

NHS ethical approval was not required as patients were not being interviewed. Ethics approval was obtained from University of Leicester and R&D approvals from participating organisations. Informed consent was obtained from all participants.

For peer review only

Results

Quantitative findings

Table 2 presents data on rates of 85+ admissions between 2007/08 and 2011/12. In improving sites these increased by 3.3% (range -2.4% to 10.6%) and in deteriorating sites by 22.7% (range 16.3% to 29.2%). During the 5 year period, the mean linear regression slope per annum was 0.003 (0.3%) for improving sites and 0.022 (2.2%) for deteriorating sites. In general, rates of admission remained fairly stable in the additional two years we examined after site selection. Between using HES data for selection and this analysis, some corrections had been made to 2007/8 data.

Changes in rates of admission for ambulatory care sensitive conditions (acute conditions such as otitis media that could normally be managed without admission and chronic conditions, such as diabetes, in which disease management could prevent exacerbations needing admission (8)) are also shown in table 2. In the three years examined, rates for acute conditions fell by 8.5% in improving sites (range -17.5% to 8.3%) and increased by 18.0% in deteriorating sites (range 4.8% to 26.8%). Mean rates of change differed less for chronic conditions, but with bigger variations between sites. They reduced by 3.9% in improving sites (range -7.4% to 0.4%) and increased by 1.0% in deteriorating sites (range -14.1% to 16.0%).

Qualitative findings

In total 142 informants contributed to either individual interviews or focus groups, as shown in table 3, which also provides a brief description of the sites The number of contributions from each site varied, in part because some agencies declined to participate. In site I2, community services and social services were provided by a single organisation. The number of informants was greater in improving than deteriorating sites (91 versus 51)

Findings are presented by 7S themes, as summarised in Table 4 Quotes are identified by site and employing organisation of respondent (PCT/CCG: A; community services: B; acute trusts: C) and respondent number. All quotes presented are from one to one interviews.

1. Strategy

This was defined as the plan of activity for the whole system, and alignment of the system to its goals. Improving sites exhibited more of a shared and comprehensive system-wide strategy for managing unplanned care, including specific policies and procedures for older people. These strategies and policies were shared across the wider health and social care system suggesting an underlying basis of collaboration and coordination, and a reduced risk of system dominance by one provider.

> *We had an audacious programme goal, which was all about reducing emergency admissions, and we had quite a lot of buy-in … to a whole-system approach. I1 A-01 (commissioning manager, CCG)*

For peer review only

For the whole time I've been here, it (urgent care) has been a top priority. We want urgent care pathways (including ambulance, ED and admissions) to be as high quality as possible. I3-A-02 (commissioning manager, CCG)

Deteriorating sites revealed less evidence of a system strategy. Although individual system components had developed strategies for aspects of unplanned care, such as reducing length of stay, there was less appreciation of how the components of the wider health system should work together. Strategies tended to be dominated by acute care provision to the detriment of policies to expand primary and community care.

The system plans have bullets like 'we'll support care close to home but we'll support financial sustainability of the acute hospitals.' Unless you have some sort of integration, those two things are mutually exclusive in the long term. D1-A-03 (service redesign manager, CCG)

An important difference between improving and deteriorating sites was their approach to improvement projects. In improving sites, these were generally well-resourced, often through funding arrangements linked to national initiatives. Moreover, they were usually given time to develop and embed into practice, rather than being subject to changing fashions or emerging policies. In contrast, in deteriorating sites projects tended to be more reactive and short-lived with little follow through.

We've piloted lots of good things, but it's been the usual story of just doing pilots and not doing them at sufficient scale, we've dabbled in things and haven't really followed them sufficiently through. D3-A-03 (commissioning director, CCG)

2. Structure

This was defined as how different components of the system related to each other. In improving sites there was closer integration of primary, acute and community services. This was facilitated by fewer organisations providing services and clearer geographical boundaries.

X was one of the few dedicated Community Trusts in the country...there's not several different providers. Patients can't get moved around the system because there's only basically GP practices, intermediate tier and one acute trust. I1-B-01 (rehabilitation manager, community trust)

The care trust... was integrated – the health organisation held the social care budget on behalf of the council ... that alone meant that the approach to commissioning was truly integrated at the budget and organisational level. So that I think is fundamental. I2-A-01 (service lead, unplanned care, CCG)

We hardly have any cross-boundary issues, it's just one social services department, one acute trust, one community trust, one mental health trust and one health commissioner – that's it. I3-A-02 (commissioning manager, CCG)

In deteriorating sites, there was less evidence of integration between acute, primary and community services. Governance and funding arrangements were more complex, with different ways of

For peer review only

working. There was often emphasis on key care stages, such as admission and discharge, but not on the wider constellation of agencies, handovers and transitions that patients face across the system.

> *The services are so complex that we as health professionals find it difficult and I think … for somebody who is over 85 or for anybody I think - it's really difficult for them to understand where to go for help and I think so by default people know that this (A and E) is open 24 hours a day, you can literally just walk in and you'll be helped. D1-C-01 (service coordinator for older people, acute trust)*

> *I don't think it [unplanned admissions], is managed very well really in this area which is one of the reasons why you see relatively high admission rates. Part of the reason for that is because there are separate organisations each with their own agendas and each with their own pressures.D2-C-01 (clinical director, unscheduled care, acute trust)*

3. Systems

This was defined as individual services contributing to the whole system. The most consistent differences between improving and deteriorating sites were in GP services, intermediate care and ED provision.

General practice

In improving sites, general practice appeared better supported financially, more innovative, for example with IT systems, and better integrated with other providers. There was closer alignment of out-of-hours GP services with either community or acute NHS providers, which facilitated closer integration of primary, acute and community services, especially for information sharing and continuity of care.

> *Round here I think GPs are more proactive in managing their patients. If you're a GMS [General Medical Services] practice there's no incentive for you keeping your admission. We had our PBC [practice-based commissioning] budgets and if we were within our budget there'd be some financial reward for that. I2-A-01 (service lead, unplanned care, CCG)*

> *(IT system) facilitates three-way conversations; the GP will be on the line, the nurse would be on the line and the acute physician from AMU [the acute medical unit] would be on the line and having a conference about whether or not it's appropriate that person ought to go to hospital. I1-A-01 (commissioning manager, CCG)*

In deteriorating sites there was a sense of under-investment and insufficient planning for primary care. GP practices were seen as providing a more limited set of services with problems of access due in part to single handed practices and half day closures, thereby increasing demand on Emergency Departments (EDs). Changes in out of hours GP provision was also thought to impact on ED attendances.

> *There's been underinvestment in primary care, there hasn't been a clear primary care strategy… there's been underinvestment in primary care local enhanced services compared to other places. D3-A-03 (commissioning director, CCG)*

For peer review only

We've got a high proportion of (older) GPs, it's I think over 50% who have retired, taken their pension and come back. So there is no motivation for them to change at all. D1-A-02 (head of development, CCG)

Intermediate Care

Intermediate care provision at scale was widely seen to be important. This was more developed at improving sites but even here, short term funding meant services were not stable. Although type of provision varied, for example the balance of home and institutionally based provision, key elements included a single point of access, a unified system often from a single provider, and multidisciplinary working to reduce duplication.

We have a single point of access manned by advice officers, behind which there are three levels of triage– at every level of triage it's an integrated triage between health and social care.I2-A-03 (strategic adviser, adult social care)

The multidisciplinary teams in intermediate care have made a difference– you know, sometimes, one person's seeing a physio, an OT and a nurse, and they just need to see one member of the team. I1- B-02 (head of reablement, community trust)

Emergency Departments

There was agreement that skill mix in ED could influence the number of older patients admitted. Initiatives in improving sites included provision of geriatricians in ED, more senior staffing and involvement of GPs. Linked to this were initiatives to provide GP with accessible alternatives to acute admission, such as rapid access clinics or telephone consultations with a geriatrician.

GPs in A & E – well GPs have been in the hospital ever since I've been here, so they've either been co-located, so very close to A & E or in A & E. I2-A-02 (commissioning manager, CCG)

We introduced our (older people's) clinic. If they (GPs) have got an elderly patient with them in the surgery, they're just not sure what to do, they just pick up this phone line, and there's a geriatrician who will advise them. I3-C-04 (director, acute and critical care, acute trust)

4. Shared values, style, staff and skills

These components are presented separately in Table 4 but considered together here as there was overlap between values and style. Additionally, some issues relating to staffing and skills have been covered in earlier sections as these seemed more relevant to specific components rather than the system as a whole (for example upskilling staff in ED). Shared values within and between components of the system appeared particularly important. Improving sites were characterised by stable staffing and leadership that supported continuity of purpose, fostered trust and collaborative working, and maintained commitment to improvement. In contrast, deteriorating sites had higher staff turnover and appeared more distracted by short term changes in policy.

It (stability) helps in terms of building those relationships and building trust and allowing us to perhaps take more risks. I2-A-02 (commissioning manager, CCG)

For peer review only

I think the things that work really well are the relationships at an operational, and to a degree going into strategic work, and I think those individual relationships, and people knowing each other and having a level of trust. I3-C-02 (commissioning manager, CCG)

No-one's in place long enough … the natural political cycle is shorter than the natural planning cycle for the health system. D3-A-01 (GP, CCG board member)

Several other values emerged from the interviews. These included a general belief that admission can be counterproductive for older people and the need to challenge a mind-set that admission is the default option.

Acute hospitals are fundamentally not the right place for over eighty fives.. you're far better in your home environment or in a supported environment that's not hospital..D3-C-01 (chief executive, acute trust)

Well, there is a culture of admission in this hospital –someone comes to the front door, they see a junior doctor, admit them. I mean, you know, if I go on a post-take ward round and see a patient that's been admitted to one of these wards on the same day and say they can be discharged, the nurse looks at me as though I'm some sort of idiot: 'What are you talking about discharge? I haven't finished admitting them yet.' D1-C-02 (clinical director, acute trust)

Several sites described a clash of values between commissioners, managers and clinicians, particularly when managing older people, and the importance of clinical leadership.

No, there's nobody clinical, there's nobody caring, there's nobody who actually does the business of looking after people, particularly the frail elderly, who are messy and don't fit into a clear protocol.I2-A-05 (GP, CCG board member)

I think what we probably need now is clinically led provider organisations cause you've still got managerial led provider organisations. The doctors do talk to each other but then they don't talk to the managers and the managers talk about a different thing so it's not necessarily connected. D1-A-02 (head of development, CCG)

For peer review only

Discussion

Summary of findings

We found some important differences between sites in which admission rates for people aged 85+ had increased most rapidly and sites in which these rates had stabilised or declined. In improving sites there was more evidence of strong strategic leadership, enabling the development of a comprehensive system-wide strategy, including specific policies and procedures for older people, which were shared across a more integrated health and social care setting. This encouraged longer-term, consistent development of strategies, often in the face of changing national imperatives. This stability also allowed trust and shared commitment to be established and the emergence of common values across the system.

In improving sites, primary care appeared stronger, both in terms of service provision and strategic engagement. This could be one reason why admissions for ambulatory care sensitive conditions fell on average in improving sites and increased in deteriorating sites. There is also evidence presented in our funder's report (18) that GP access was better in improving sites. Intermediate care was also more developed in improving sites. These services appeared to work best when provided at scale and fully integrated with each other, offering round-the-clock availability with a single point of access, shared information systems and specialist nursing and geriatric support. Improving sites also seemed more equipped to reduce admissions of older people from ED, through a variety of initiatives, including more senior staffing, involvement of GPs and provision of specialist nurses or geriatricians. In summary, improving sites generally made fuller use of a suite of strategies to reduce unplanned admissions, and importantly had a more system-wide outlook and strategic approach.

For peer review only

Comparison with previous studies

Findings from several recent qualitative and mixed methods studies are consistent with those reported here. A study examining six emergency and urgent care sites suggested that improving GP out of hours access, senior review in ED and multidisciplinary teams could reduce admissions(27), and others have emphasised how the culture and staffing in ED can influence admission rates (28, 29). We are not aware of previous qualitative work examining the whole system, but our findings support recommendations on integrated care made by the Kings Fund, including 'sharing sovereignty', developing a persuasive vision and establishing leadership.(30). More recently a CQC report concluded: 'To truly coordinate care, local system leaders must ensure there is a golden thread linking vision to delivery, so that everyone involved can not only share the vision but see themselves as part of the team that delivers it'.(31)

Strengths and limitations

Our study design offered strong internal validity through in-depth analysis within case and structured comparison between cases.(32) The design and conduct of this study reflects a number of features to enhance trustworthiness.(33). The approach allowed a common method of data collection and analysis subject to open scrutiny by the wider research team and advisors, enhancing dependability. In terms of confirmability, the researchers were supported in being reflexive about their own role in data collection, including review meetings with the wider research team.

That researchers and participants knew how each site had performed helped to inform the interviews; by presenting data on admissions we were able to engage in a more detailed discussion of issues and strategies. It is unlikely that informants would be unaware of their own performance. However this approach may have meant that both interviewers and participants may have focused on what was perceived to be working well in improving sites, and on more negative issues in deteriorating sites. In retrospect, blinding some rounds of data analysis to categorisation of sites may have reduced the risk of bias.

The study had several other limitations. Our selection criteria were based on changes in historic data; the additional data on admissions for 2010/11 and 2011 showed that over the five year period

differences between improving and deteriorating sites persisted but in most sites stabilised somewhat over the last two years, and the trajectory of improvement or deterioration slowed. As with any study of outliers there is a possibility that some changes represented regression to the mean. Furthermore there were differences in performance within improving and deteriorating groups: in sites I1 and I3 the five year trend showed a small increase in admission rates, although less than in deteriorating sites. . Some informants found it challenging to reflect on past events rather than the current situation. The strongest interview data came from informants who had been in post for the period of interest and so able to provide an institutional memory. This problem was compounded by the fact the study was conducted during a period of organisational upheaval in the NHS, leading to many informants being relatively new in post. Finally, the snowball sampling technique may have led to under-representation of some groups, such as ambulance services.

The pace of change within the NHS has quickened since the study was conducted, with additional challenges imposed by austerity. However, we feel our key messages remain relevant as they emphasise the need for strategies and stability that can weather the impact of ongoing changes. We also acknowledge that sites with a more complex mix of acute trusts (which we deliberately excluded) may experience different or additional problems and solutions.

The McKinsey 7S framework was effective in enabling the systematic investigation of system components and their interaction, but was less useful in mapping more abstract issues such as style and values. The framework appeared better suited to examining individual organisations, rather than large complex systems of inter-dependent heterogeneous system actors, which may have been further understood and analysed using, for example, Beer's Viable Systems Model (22).

Implications for policy makers and service providers

This study supports taking a whole system approach in designing services for older people. This is best achieved by developing a sustained and shared vision focusing on outcomes that matter to older people(12), establishing strong leadership without dominance by one organisation, maximising integration and minimising complexity within the system. These findings have potential to inform the practical implementation in England of Strategic Transformation Partnerships and Accountable Care Organisations, vehicles designed to promote system-based collaboration. (34)

Conclusions

Health and social care communities which attenuated unplanned admissions of people aged 85+ prioritised developing a shared vision and strategy, encompassing multiple organisations and backed by strong leadership and shared values. This allowed sustained implementation of a suite of interventions, including better-developed primary and intermediate care services working closely with the hospital and emergency departments.

For peer review only

Data sharing

Full transcripts are available from the corresponding author.

Contributions

All authors contributed to study design, and analysis, and have approved the final manuscript. Andrew Wilson led the study, Richard Baker and Stephen Rogers contributed expertise in primary care and quality improvement, John Bankart and Stoyan Kurtev led on selection of sites. Jay Banerjee and Simon Conroy constructed expertise on ED policies and geriatric medicine respectively. Ran Bhamra contributed expertise on system theory and Justin Waring led the analysis. Kay Phelps and Emma Regen led on fields work and conducted most of the interviews.

Acknowledgements

We are grateful for the support of members of the steering group throughout the project, and for advice from the Leicestershire, Northamptonshire and Rutland Older People's Research Patient and Public Involvement Forum. Access to sites was facilitated by the NIHR Clinical Research network. Most of all we would like to acknowledge participants at all study sites for their time and the candid insights they provided.

Conflict of interest

The authors declared no potential conflicts of interest with respect to the research, authorship and/or publication of this article.

Funding

The project was funded by the NIHR Health Service and Delivery and Research Programme (project number 10/1010/05). The views and opinions expressed in this article are those of the authors and do not necessarily reflect those of the Health Service and Delivery and Research Programme, NIHR, NHS or the Department of Health.

For peer review only

References

1. Bobrovitz N, Onakpoya I, Roberts N, Heneghan C, Mahtani KR. Protocol for an overview of systematic reviews of interventions to reduce unscheduled hospital admissions among adults. BMJ open. 2015;5(8):e008269-e.
2. García-Pérez L, Linertová R, Lorenzo-Riera A, Vázquez-Díaz JR, Duque-González B, Sarría-Santamera A. Risk factors for hospital readmissions in elderly patients: a systematic review. QJM: An International Journal of Medicine. 2011;104(8):639-51.
3. Tang N, Stein J, Hsia RY, Maselli JH, Gonzales R. Trends and Characteristics of US Emergency Department Visits, 1997-2007. JAMA. 2010;304(6):664-70.
4. Schuur JD, Venkatesh AK. The Growing Role of Emergency Departments in Hospital Admissions. New England Journal of Medicine. 2012;367(5):391-3.
5. Raphael Wittenberg LS, Barry McCormick, Jeremy Hurst. Understanding emergency hospital admissions of older people. Oxford: 2014.
6. Purdy S. Avoiding hospital admissions - What does the research evidence say? London: Kings Fund, 2010.
7. University of Birmingham Health Services Management Centre. Reducing Unplanned Hospital Admissions: what does the literature tell us? Birmingham: 2006.
8. Tian Y, Dixon A, Gao H. Emergency hospital admissions for ambulatory care sensitive condtions: identifying the potential for reducations. London: The Kings Fund, 2012.
9. O'Cathain A, Knowles E, Maheswaran R, Pearson T, Turner J, Hirst E, et al. A system-wide approach to explaining variation in potentially avoidable emergency admissions: national ecological study. BMJ Quality & Safety. 2014;23(1):47-55.
10. Sager MA, Franke T, Inouye SK, Landefeld CS, Morgan TM, Rudberg MA, et al. Functional Outcomes of Acute Medical Illness and Hospitalization in Older Persons. Arch Intern Med. 1996;156(6):645-52.
11. Landeiro F, Roberts K, Gray AM, Leal J. Delayed Hospital Discharges of Older Patients: A Systematic Review on Prevalence and Costs. The Gerontologist. 2017:gnx028.
12. Akpan A, Roberts C, Bandeen-Roche K, Batty B, Bausewein C, Bell D, et al. Standard set of health outcome measures for older persons. BMC Geriatrics. 2018;18(1):36.
13. Shepperd S, Iliffe S, Doll H, Clarke M, Kalra L, Wilson AD, et al. Admission avoidance hospital at home. Cochrane Database of Systematic Reviews [Internet]. 2016; (9). Available from: http://onlinelibrary.wiley.com/doi/10.1002/14651858.CD007491.pub2/abstract;jsessionid=D3133E6EEEA6E31B6A45F33C66800223.f03t02
14. NHS Benchmarking Network. National Audit of Intermediate Care. Manchester: NHS Benchmarking Network, 2013.
15. Barker I, Steventon A, Deeny SR. Association between continuity of care in general practice and hospital admissions for ambulatory care sensitive conditions: cross sectional study of routinely collected, person level data. BMJ. 2017;356.
16. White AL, Armstrong PAR, Thakore S. Impact of senior clinical review on patient disposition from the emergency department. Emergency Medicine Journal. 2010;27(4):262-5.
17. Damery S, Flanagan S, Combes G. Does integrated care reduce hospital activity for patients with chronic diseases? An umbrella review of systematic reviews. BMJ Open. 2016;6(11).
18. Wilson A, Baker R, Bankart J, Banerjee J, Bhamra R, Conroy S, et al. Establishing and implementing best practice to reduce unplanned admissions in those 85 years and over through system change (Establishing System Change for Admissions of People 85+(ESCAPE 85+)): a mixed-methods case study approach. Health Services and Delivery Research. 2015;3:37.
19. Yin RK. Multiple case studies with embedded sub cases. Case Study Research: Design and Methods. Fourth Edition ed. California: Sage Publications; 2009.
20. NHS_Digital. Hospital Episode Statistics.

For peer review only

21. NHS_digital. Emergency hospital admissions: acute conditions usually managed in primary care: indirectly standardised rate, all ages, annual trend, F,M,P 2016 [cited 15/05/2018]. Available from: https://digital.nhs.uk/data-and-information/publications/clinical-indicators/compendium-of-population-health-indicators/compendium-hospital-care/current/emergency-admissions/emergency-hospital-admissions-acute-conditions-usually-managed-in-primary-care-indirectly-standardised-rate-all-ages-annual-trend-f-m-p.
22. Purdy S, Griffin T, Salisbury C, Sharp D. Ambulatory care sensitive conditions: terminology and disease coding need to be more specific to aid policy makers and clinicians. Public Health. 2009;123(2):169-73.
23. Marshall MN. The key informant technique. Family Practice. 1996;13(1):92-7.
24. Gale N, Heath G, Cameron E, Rashid S, Redwood S. Using the framework method for the analysis of qualitative data in multi-disciplinary health research. BMC Medical Research Methodology. 2013;13(1):117.
25. NVivo qualitative data analysis software. QSR International Pty Ltd Version 7. 2006.
26. Boeije H. A purposeful approach to the constant comparative method in the analysis of qualitative interviews. Qual Quant. 2002;36(4):391 - 409.
27. O'Cathain A, Knowles E, Turner J, Hirst E, Goodacre S, Nicholl J. Variation in avoidable emergency admissions: multiple case studies of emergency and urgent care systems. Journal of Health Services Research & Policy. 2016;21(1):5-14.
28. Pinkney J RS, Benger J, Brant H, Joel-Edgar S, Swancutt D, et al. How can frontline expertise and new models of care best contribute to safely reducing avoidable acute admissions?

A mixed-methods study of four acute hospitals. Health Serv Deliv Res. 2016;4(3).
29. Pope I, Burn H, Ismail SA, Harris T, McCoy D. A qualitative study exploring the factors influencing admission to hospital from the emergency department. BMJ Open. 2017;7(8).
30. Ham C, Walsh N. Making integrated care happen at scale and pace. London: The Kings Fund; 2013.
31. Commission CQ. The state of health care and adult social care in England 2016/17. London: Care Quality Commission 2017.
32. Mintzberg H, Lampel JB, Quinn J, Ghoshal S. The Strategy Process: Concepts, Contexts, Cases, 5/E. Harlow: Pearson; 2014.
33. Lincoln YS, Guba EG. Establishing trustworthiness. London: Sage; 1985. 289-331 p.
34. NHS_Providers. STPs and accountable care: background briefing. 2018.

For peer review only

PCT Rank for slope (n=143)	85+ admission rate (number of admissions/100 population aged 85+)			Slope (per annum change)	% change in rate	% admissions to linked hospital trust	% aged 85+	Reference in paper
	2007/08	2008/09	2009/10					
4	0.55	0.51	0.51	-0.02	-7.3	89	2.6	I1
5	0.61	0.6	0.57	-0.02	-6.6	87	2.6	I3
9	0.41	0.41	0.39	-0.01	-4.9	83	2.2	I2
132	0.48	0.54	0.59	0.06	22.9	92	2.2	D1
133	0.41	0.45	0.52	0.06	26.8	87	1.7	D3
135	0.49	0.59	0.61	0.06	25.5	83	1.8	D2

Table 1: Selection of improving (I) and deteriorating (D) Primary Care Trusts (PCT)

For peer review only

	I1	I2	I3	D1	D2	D3		Improving sites average *	Deteriorating sites average *
**85+ admissions/100 aged 85+ per annum**									
2007/08	0.47	0.41	0.57	0.48	0.49	0.40		0.48	0.46
2008/09	0.51	0.42	0.6	0.55	0.59	0.45		0.51	0.53
2009/10	0.51	0.40	0.58	0.60	0.61	0.53		0.50	0.58
2010/11	0.54	0.39	0.58	0.60	0.56	0.48		0.50	0.55
2011/12	0.52	0.40	0.58	0.62	0.57	0.49		0.50	0.56
% change between 2007/8 and 2011/12	10.64	-2.44	1.75	29.17	16.33	22.50		3.32	22.67
Linear regression slope (per annum change in rate)	0.013	-0.005	0	0.033	0.013	0.020		0.003	0.022
**Acute ACSC all ages. Indirectly age and sex standardised rate per 100,000**									
2007/08	424	579	462	367	397	619		488	461
2008/09	515	591	347	388	461	699		484	516
2009/10	350	627	387	449	416	785		455	550
% change between 2007/8 and 2009/10	-17.45	8.29	-16.23	22.34	4.79	26.82		-8.46	17.98
Linear regression slope (per annum change in rate)	-37	24	-37.5	41	9.5	83		-16.83	44.50
**Chronic ACSC all ages. Indirectly age and sex standardised rate per 100,000**									
07/08	220	240	148	188	177	247		203	204
08/09	276	247	104	212	177	265		209	218
09/10	210	241	137	218	152	249		196	206
% change between 2007/8 and 2009/10	-4.55	0.42	-7.43	15.96	-14.12	0.81		-3.85	0.88
Linear regression slope (per annum change in rate)	-5	0.5	-5.5	15	-12.5	1		-3.33	1.17

For peer review only

Table 2: Changes in admission rates for patients aged 85+, and rates of admission for acute and chronic ambulatory care sensitive conditions (ACSC) in improving and deteriorating sites

*Averages derived from rows not columns.

For peer review only

Sites	Description					Participants					
		ONS classification	Ranking of population size (151 PCTs, 1 = largest)	Deprivation rank (151 PCTs, 1 = most deprived)	Acute provision	Acute Trust	PCT/ CCG	Community Services	Social services	PPI	Total
I1	Major city	Regional centre	85	56	1 university hospital, 1 district general hospital	declined	2	13 (7 individual, 2 focus groups [n=2 and 4])	declined		15
I2	largely rural area comprising three small to medium-sized towns.	Manufacturing town	139	40	3 district general hospitals	6	6	16 (7 individual, 2 focus groups [n=9])		Focus group (n= 5)	33
I3	semirural and urban conurbation in close proximity to metropolitan area.	Industrial hinterlands	42	50	2 district general hospitals	5	3	24 (4 individual, 3 focus groups [n=6,6,8])	2	Focus group (n=9)	43

For peer review only

D1	Major city	Centre with industry	56	43	1 large acute hospital	7	3	3	2	Focus group (n=5)	20
D2	three small to medium-sized towns	Centre with industry	120	22	4 district general hospitals	10	3	2	4	Focus group (n=5)	24
D3	mixed urban and rural area	New and growing town	118	119	1 large acute hospital	2	3	declined	1	1	7
Total						30	20	58	9	25	142

Table 3: Description of sites and informants by organisational category

For peer review only

	7S						
	Strategy	Structure	Systems	Shared values	Skills	Style	Staff
I1	High levels of investment in community provision.<br>Early engagement of all stakeholders in strategy development	Strong linkages between hospital, GP and community care<br>Small number of large providers	Innovative cross-sectoral technology systems<br>Unified provision of intermediate care	Professionals willing to work together and bend hierarchies to reach shared goals<br>Pride in in providing services to keep older people out of hospital	Staff have perseverance and skills to see through projects and get people on board	Regular contacts between hospital and community providers	Effective multidisciplinary teams in intermediate care<br>Recognised need to involve pharmacists to reduce excessive medication
I2	Care trust developed strategy across social and community health services	Integrated budget for commissioning health and social care	Focus on practice based commissioning providing incentives for GPs to reduce admissions<br>Single point of access 24/7 for intermediate care	Strong and stable organisational cohesion	High levels of interpersonal skills enable effective working relationships	Close links between GPs and other service providers	Longstanding and close working relationships
I3	Urgent care a top strategic priority<br>Strategy recognises the importance of community care	Lack of boundary issues helps maintain a clear structure, with small number of providers	Out of hours run by through community trust<br>Rapid access to clinics and telephone consultations with geriatricians	Strong organisational cohesion	High levels of skills in community teams	Some 'blame culture' when services are pressurised	
D1	Frequent changes in leadership	Complex structures for health community care	Perception that GPs were demotivated	Historically poor relationship between	Perception that care	Culture of admission	Recognised shortage of geriatricians

For peer review only

	roles and regional strategies<br>Acute Trust dominant in determining strategy		Lack of well-functioning multi-disciplinary teams	Trusts and community providers	home staff are under skilled	from ED as default option	
D2	Lack of clear strategy on unplanned admissions; more focus on reducing length of stay than admission avoidance	Poor integration between primary and secondary care, and between ambulance services and acute Trust	Frequent restructuring of intermediate care	Lack of shared culture between organisations	Recognised need for skilled geriatricians in acute medical unit	Recent focus on clinical leadership	Lack of senior medical staff in ED<br>Recent investment in geriatricians and community matrons
D3	More focus on elective care than urgent care<br>Successful pilots not followed through	Frequent changes in structure of system, including hospital sites and structure of intermediate care	Low investment in primary care	Conflict between medical, rehabilitation and managerial values	Perception that insufficient staff have skills needed to assess frail elderly	Recent focus on clinical leadership	Inadequate provision of community matrons

Table 4: Features of sites by McKinsey 7S categories

For peer review only

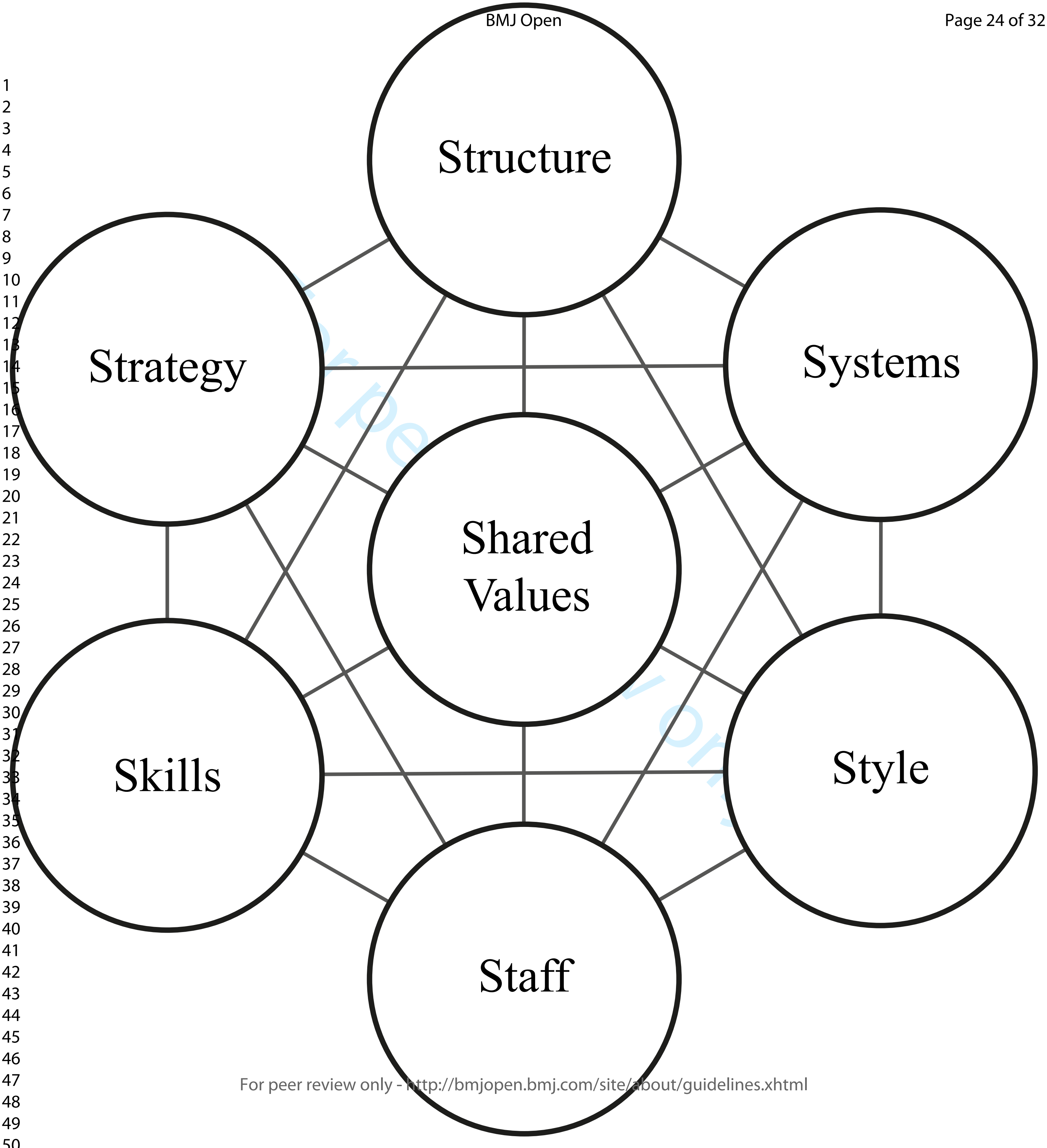
Structure
Strategy
Systems
Shared Values
Skills
Style
Staff

Figure 1: The McKinsey 7S Framework and how it was applied

Strategy: the plan of activity for the whole system, and alignment of the system to its goals

Structure: how different components of the system related to each other.

Systems: individual services contributing to the whole system.

Shared values: the norms and standards that guide the behaviour of the human elements within the system.

Style: the style of management used by the system leadership.

Staff: training, motivation and rewards of the staff.

Skills: specific skills existing and required by staff in order to best execute their duties.

For peer review only

Appendix 1: detailed topic guide

Unplanned admissions in the area: priorities, levels and changes Ask interviewee to give a reaction to the data presented to them giving the rates of unplanned admissions of people 85+ in their area during 2005–9.

Historical perspective from 2005 onwards.

Possible questions and prompts:

(a) Were you aware of the rate of unplanned admissions to hospital of people 85+ in your area? (b) Did you know if your rates were increasing decreasing or staying the same? – What would you say were the reasons for that?

(c) Are the rates of unplanned admissions to acute hospital of people 85+ an issue in your organisation? – Do they need to be reduced?

(d) Do you collect/collate any local data on unplanned admissions and if so can we see the reports/have access to the data?

Whole system characteristics, configuration, monitoring and changes over time What are the important elements/components which you believe have the most effect on the rates of unplanned admissions of people 85+ in your area?

Possible prompts (could use star model to illustrate):

(a) Policies. – Procedures, service delivery, working practices.

(b) People, staffing, leadership, behaviour. (c) Finance and budgets. (d) Infrastructure, geography, buildings, IT. (e) Partner organisations. (f) Shared vision.

Possible questions:

(a) What characteristics are associated with changes in unplanned admissions of people 85+? (b) What changes have you made as a result of the increasing number of people over 85? (c) What changes do you think you need to make to reduce unplanned admissions of people 85+? (d) What changes are planned to reduce unplanned admissions of people 85+? (e) What have been the historical drivers of change? – Leadership. – Finance. – Targets.

(f) When making changes what have been the main challenges? (g) What have been the main outcomes of change? – Did it achieve what was hoped for?

(h) Reasons for success or failure of changes.

APPENDIX 2

NIHR Journals Library www.journalslibrary.nihr.ac.uk

104

Policy on unplanned admissions

(a) How have/do policies in your organisation affect unplanned admissions of people aged 85+? – Historical perspective as well as current. – E.g. A&E, ED, Primary Care, out-of-hours services, Ambulance, Staffing.

For peer review only

(b) What have been/are your organisation's policies around unplanned admissions? – Historical perspective as well as current.

(c) Have policies around unplanned admissions been linked/coherent with the policies of other organisations in your area? – Historical perspective as well as current.

(d) What policy changes have you made as a result of the increasing number of people over 85? (e) Have there been/are there clear strategies/goals within your organisation around unplanned admissions of people 85+? (f) Please can you supply policy documentation on unplanned admissions of people 85+ from 2005 to the present?

Budgets/finance/commissioning of emergency care

(a) Is there a specific budget for unplanned/emergency care for people aged 85+/older people? – Historically and currently.

(b) How much is spent on emergency care for people aged 85+? – Historically and currently.

(c) How much is spent on older people compared with other areas? – Historically and currently.

(d) What are the commissioning arrangements for unplanned admissions (of people 85+) in your area? – CCGs. – PCTs. – Involvement of secondary care.

(e) How have these arrangements changed since 2005? (f) What part have financial imperatives (cost/resources) played in commissioning services for unplanned admissions? (g) What are the incentives in commissioning which affect unplanned admissions?

DOI: 10.3310/hsdr03370 HEALTH SERVICES AND DELIVERY RESEARCH 2015 VOL. 3 NO. 37

© Queen's Printer and Controller of HMSO 2015. This work was produced by Wilson et al. under the terms of a commissioning contract issued by the Secretary of State for Health. This issue may be freely reproduced for the purposes of private research and study and extracts (or indeed, the full report) may be included in professional journals provided that suitable acknowledgement is made and the reproduction is not associated with any form of advertising. Applications for commercial reproduction should be addressed to: NIHR Journals Library, National Institute for Health Research, Evaluation, Trials and Studies Coordinating Centre, Alpha House, University of Southampton Science Park, Southampton SO16 7NS, UK.

105

Partnership working between acute and community health and social care

(a) How have acute and community health and social care worked together in the area to tackle unplanned admissions and care of older people? (b) Has there been a whole systems approach to planning care or do all the organisations commission, plan and deliver service separately? E.g. – Cooperative interorganisational networks. – Sharing information and communication. – Shared protocols and procedures. – Shared policy on unplanned admissions – linked/coherent?

(c) Have there been any integrated services or is this planned? (d) What have been the incentives to a whole systems approach? (e) What have been the challenges to a whole systems approach and how does cooperation/competition pose challenges? (f) Do the health- and social care organisations in your area have clearly defined boundaries? – What are the boundaries? – Are boundaries a problem? – Different measurement criteria for success? – Different organisational cultures? – Different regulation?

For peer review only

(g) Outcomes of partnership/integrated planning/services? – Successes and challenges. – Reasons for success or failure. – Obtain copies of any partnership agreements.

People, leadership, managerial and clinical relationships, staffing

(a) Who has led policy and/or changes affecting unplanned admissions of people over 85 in this area? – Organisational leadership.

(b) Have there been any professional tensions, allegiances and hierarchies which affect unplanned admissions in this area? (c) Have there been any staff satisfaction surveys carried out in your organisation? – Can we have report?

(d) Have job planning and staffing changes had an impact on unplanned admissions? (e) What impact has training had on unplanned admissions?

Infrastructure and contextual factors influencing unplanned admissions of 85+

(a) Have there been any local infrastructure or contextual factors or pressures influencing unplanned admissions of 85+ in this area? Possible prompts: – Geography. – Buildings/facilities. – Technology/IT systems. – Transport – ambulance services. – Environmental conditions. – Local pressures.

APPENDIX 2

NIHR Journals Library www.journalslibrary.nihr.ac.uk

106

Procedures/service delivery

(a) Are there any issues around service delivery in your area which have affected unplanned admissions of people 85+? Possible prompts: – Who delivers urgent care services in the acute sector? – Who delivers urgent care services in the community sector? – Commissioning arrangements. – Pathways of care. – Access points for urgent and emergency care. – Admission pathways. – Management of acute care. – ED configuration. – Integrated care. – Access to and responsiveness of social care. – Management of chronic conditions. – Out-of-hours provision in the community. – Ambulance service. – Discharge procedures. – IT systems (Dashboard, NHS Pathways, discharge monitoring systems, etc.). – Governance systems (emergency care networks, joint governance frameworks).

Interventions, outcomes and evaluation

(a) What interventions in the whole system have affected or changed the levels of unplanned admissions? – Intermediate care. – Integrated care. – Reablement – End of life care. – Residential/nursing home care. – Community matrons. – Alternatives to acute admission. – Discharge initiatives. – Social care initiatives. – Chronic conditions management. – Geriatrician assessment/outreach. – Hospital outreach. – Hospice care/outreach.

(b) Who delivers these services? (c) What has been the effect of these interventions on the whole system of care? (d) What is the relationship between interventions? (e) How available and accessible are alternatives to unplanned admission? – E.g. primary and social care services.

(f) Have the outcomes of the interventions been measured or evaluated? (g) What are the reasons for the success of these interventions? (h) What are the reasons for the failure of these interventions? (i) Can I have copies of any internal evaluations?

For peer review only

DOI: 10.3310/hsdr03370 HEALTH SERVICES AND DELIVERY RESEARCH 2015 VOL. 3 NO. 37

© Queen's Printer and Controller of HMSO 2015. This work was produced by Wilson et al. under the terms of a commissioning contract issued by the Secretary of State for Health. This issue may be freely reproduced for the purposes of private research and study and extracts (or indeed, the full report) may be included in professional journals provided that suitable acknowledgement is made and the reproduction is not associated with any form of advertising. Applications for commercial reproduction should be addressed to: NIHR Journals Library, National Institute for Health Research, Evaluation, Trials and Studies Coordinating Centre, Alpha House, University of Southampton Science Park, Southampton SO16 7NS, UK.

Recommendations for reducing emergency admissions

(a) What changes have been made/need to be made to reduce unplanned admissions? – Strategy. – Organisational. – Partnership. – Commissioning. – Policy. – Service delivery. – Leadership/staffing.

(b) What lessons have you learnt in trying to reduce unplanned admissions for those 85+? – What are the challenges? – What have been the successes?

PPI involvement

(a) What PPI involvement do you have in your organisation in this area? (b) What PPI involvement do you have in policy making in this area? (c) What PPI involvement do you have in commissioning in this area? (d) What PPI involvement do you have in strategy development in this area? (e) What PPI involvement do you have in system development in this area? (f) What PPI involvement do you have in leadership and decision making in this area? (g) What PPI involvement do you have in service delivery in this area? (h) What changes have resulted from PPI involvement?

Outcomes and impact for older people and carers

(a) How do you measure outcomes for older people and carers? (b) What service changes have most affected older people and carers in this area? – What has been the impact?

(c) What has been the impact of particular initiatives to reduce unplanned admissions? – Refer to initiatives already mentioned above. – What is the patient experience of these services?

(d) Have outcomes for older people and carers changed over the last 5 years? – For the better? – For the worse?

(e) What changes are needed to improve emergency services and outcomes for older people 85+ and carers? (f) What are the benefits to older people and their carers of reducing unplanned admissions? (g) What are the disadvantages to older people and their carers of reducing unplanned admissions? (h) Have you carried out any patient satisfaction surveys in emergency/unplanned/urgent care in the last 5 years? – Can we have the report?

(i) Have you carried out any service evaluation looking at urgent care services for people over 85?

For peer review only

Consolidated criteria for reporting qualitative studies (COREQ): 32-item checklist

Developed from:
Tong A, Sainsbury P, Craig J. Consolidated criteria for reporting qualitative research (COREQ): a 32-item checklist for interviews and focus groups. *International Journal for Quality in Health Care*. 2007. Volume 19, Number 6: pp. 349 – 357

No. Item	Guide questions/description	Reported on Page #
**Domain 1: Research team and reflexivity**		
Personal Characteristics		
1. Inter viewer/facilitator	Which author/s conducted the inter view or focus group?	4
2. Credentials	What were the researcher's credentials? E.g. PhD, MD	4
3. Occupation	What was their occupation at the time of the study?	4
4. Gender	Was the researcher male or female?	Author list
5. Experience and training	What experience or training did the researcher have?	4
Relationship with participants		
6. Relationship established	Was a relationship established prior to study commencement?	no
7. Participant knowledge of the interviewer	What did the participants know about the researcher? e.g. personal goals, reasons for doing the research	no
8. Interviewer characteristics	What characteristics were reported about the inter viewer/facilitator? e.g. Bias, assumptions, reasons and interests in the research topic	no

For peer review only

**Domain 2: study design**		
Theoretical framework		
9. Methodological orientation and Theory	What methodological orientation was stated to underpin the study? e.g. grounded theory, discourse analysis, ethnography, phenomenology, content analysis	3
Participant selection		
10. Sampling	How were participants selected? e.g. purposive, convenience, consecutive, snowball	3
11. Method of approach	How were participants approached? e.g. face-to-face, telephone, mail, email	3
12. Sample size	How many participants were in the study?	4
13. Non-participation	How many people refused to participate or dropped out? Reasons?	4
Setting		
14. Setting of data collection	Where was the data collected? e.g. home, clinic, workplace	4
15. Presence of non-participants	Was anyone else present besides the participants and researchers?	no
16. Description of sample	What are the important characteristics of the sample? e.g. demographic data, date	Table 2
Data collection		
17. Interview guide	Were questions, prompts, guides provided by the authors? Was it pilot tested?	4
18. Repeat interviews	Were repeat inter views carried out? If yes, how many?	no
19. Audio/visual recording	Did the research use audio or visual recording to collect the data?	4
20. Field notes	Were field notes made during and/or after the inter view or focus group?	4
21. Duration	What was the duration of the inter views or focus group?	4
22. Data saturation	Was data saturation discussed?	
23. Transcripts returned	Were transcripts returned to participants for comment and/or correction?	no
**Domain 3: analysis and findings**		
Data analysis		

For peer review only

24. Number of data coders	How many data coders coded the data?	4
25. Description of the coding tree	Did authors provide a description of the coding tree?	4
26. Derivation of themes	Were themes identified in advance or derived from the data?	4
27. Software	What software, if applicable, was used to manage the data?	4
28. Participant checking	Did participants provide feedback on the findings?	no
Reporting		
29. Quotations presented	Were participant quotations presented to illustrate the themes/findings? Was each quotation identified? e.g. participant number	throughout
30. Data and findings consistent	Was there consistency between the data presented and the findings?	throughout
31. Clarity of major themes	Were major themes clearly presented in the findings?	throughout
32. Clarity of minor themes	Is there a description of diverse cases or discussion of minor themes?	Minor themes

For peer review only

BMJ Open

Understanding variation in unplanned admissions of people aged 85 and over: a systems-based approach

Journal:	*BMJ Open*
Manuscript ID	bmjopen-2018-026405.R2
Article Type:	Research
Date Submitted by the Author:	20-May-2019
Complete List of Authors:	Wilson, Andrew; University of Leicester, Health Sciences<br>Baker, Richard; University of Leicester, Health Sciences<br>Bankart, John; University of Leicester, Health Sciences<br>Banerjee, J; University Hospitals of Leicester NHS Trust, Infirmary Square, Leiceste<br>Bhamra, Ran; Loughborough University<br>Conroy, Simon; University of Leicester<br>Kurtev, Stoyan; University of Leicester, Health Sciences<br>Phelps, Kay; University of Leicester College of Medicine Biological Sciences and Psychology, Health Sciences<br>Regen, Emma; University of Leicester College of Medicine Biological Sciences and Psychology, Health Sciences<br>Rogers, Stephen; University of Leicester, Department of Health Sciences<br>Waring, Justin; University of Nottingham, Centre for Health Innovation, Leadership & Learning / Nottingham University Business School
**Primary Subject Heading**:	Geriatric medicine
Secondary Subject Heading:	Health services research
Keywords:	Older people, Unplanned admissions, Health systems, Mixed methods

SCHOLARONE™
Manuscripts

Understanding variation in unplanned admissions of people aged 85 and over: a systems based approach

Andrew Wilson (1), Richard Baker (1), John Bankart (1), Jay Banerjee (2), Ran Bhamra (3), Simon Conroy (1), Stoyan Kurtev (1), Kay Phelps (1), Emma Regen (1), Stephen Rogers (4) and Justin Waring (5)

(1) Department of Health Sciences, University of Leicester, Leicester, UK
(2) University Hospitals of Leicester NHS Trust, Leicester, UK
(3) Wolfson School of Mechanical, Electrical and Manufacturing Engineering, Loughborough University, Loughborough, UK
(4) Department of Public Health, NHS Northamptonshire, Northampton, UK
(5) Business School, University of Nottingham, Nottingham, UK

Corresponding author

Professor Andrew Wilson
Department of Health Sciences
College of Life Sciences
University of Leicester, George Davies Centre
Leicester LE1 7RH

aw7@le.ac.uk

For peer review only - http://bmjopen.bmj.com/site/about/guidelines.xhtml

Understanding variation in unplanned admissions of people aged 85 and over: a systems-based approach

Abstract

Aim: To examine system characteristics associated with variations in unplanned admission rates in those aged 85+.

Design: Mixed methods

Setting: Primary Care Trusts in England were ranked according to changes in admission rates for people aged 85+ between 2007 and 2009, and study sites selected from each end of the distribution: three 'improving' sites where rates had declined by more than 4% and three 'deteriorating' sites where rates had increased by more than 20%. Each site comprised an acute hospital trust, its linked primary care trust/clinical commissioning group, the provider of community health services, and adult social care.

Participants: A total of 142 representatives from these organisations were interviewed to understand how policies had been developed and implemented. McKinsey's 7S framework was used as a structure for investigation and analysis.

Results: In general, improving sites provided more evidence of comprehensive system focused strategies backed by strong leadership, enabling the development and implementation of policies and procedures to avoid un-necessary admissions of older people. In these sites, primary and intermediate care services appeared more comprehensive and better integrated with other parts of the system, and policies in Emergency Departments were more focused on providing alternatives to admission.

Conclusions: Health and social care communities which have attenuated admissions of people aged 85+ prioritised developing a shared vision and strategy, with sustained implementation of a suite of interventions.

For peer review only - http://bmjopen.bmj.com/site/about/guidelines.xhtml

Strengths and limitations of this study'

- In England, unplanned hospital admissions for the people aged 85 and over are rising but there is substantial geographical variation.
- A 'whole system approach' can be used to understand this variation between health economies.
- Through qualitative interviews with a comprehensive range of informants, we examined three sites where rates of admission in this age group rose most sharply and three in which the rise was reversed or attenuated.
- The study relied on institutional memory at a time of transition.

For peer review only

Key words

Older people

Unplanned admissions

Health systems

Mixed methods

Understanding variation in unplanned admissions of people aged 85 and over: a systems-based approach

Word count: 4602

Introduction

Internationally, unplanned hospital admissions have increased steadily over recent decades.(1-4) In England between 2001/2 and 2012/13, unplanned admissions of people aged 65+ rose by 46% and the age-standardised rate by 25%. Rates of increase rose steadily with age: from 9.9% for those aged 65-69 to 50.2% for those aged 90+.(5)

Research consistently finds unexplained variations in the rise of unplanned admissions, suggesting lessons may be learned from different experiences.(6-9) Alternatives to admission are particularly important in those aged 85+, who often present with multiple comorbidities, polypharmacy, cognitive impairment and disability. Once admitted, they have longer stays, are more prone to hospital acquired complications, and may experience more difficulty returning to their usual place of residence.(10, 11) Furthermore, minimising time spent in hospital is a health outcome that matters to older people.(12) There is increasing evidence that alternatives to acute admission, such as Hospital at Home, produce similar if not better outcomes (13), although in England, provision of these services remains about half what is needed.(14)

Several initiatives have been introduced to address the increase in acute admissions.(6) There is good evidence that higher continuity in primary care is associated with fewer admissions(15), and that senior review in Emergency Departments (ED) can be effective in reducing admissions.(16) There is also some evidence of benefit from integrating primary and secondary care and health and social care.(17) However as the Kings Fund report concluded, 'a combination of interventions intended to reduce admissions may be expected to have a 'cumulative' effect'.(6) To understand variations in admissions it is therefore useful to examine the configuration of the whole system.

As most of the variation in admission rates is due to relatively immutable demographic factors such as deprivation(6), examining differing trends in rates over time provides an opportunity to explore the impact of changes in service configurations.

In this study, we aimed to identify system characteristics associated with higher and lower increases in unplanned admission rates in those aged 85+. This article focuses on the qualitative component of a mixed method study reported fully elsewhere.(18)

Methods

Methodology

We used a qualitative, multiple, explanatory case study approach as our principal method.(19) Following consideration of tools to enable the analysis of complex organisations, the McKinsey 7S framework was selected as shown in Figure 1 [20] Its particular contribution was to help understand

For peer review only - http://bmjopen.bmj.com/site/about/guidelines.xhtml

the inherent complexity of the system as a whole, and emphasise that for change to be effective, changes in any one component should be accompanied by complementary changes in others.

Site selection

Six study sites were selected based on admission data for patients aged 85+ from English primary care trusts (PCTs). These organisations were responsible for commissioning primary, community and secondary health services, and were succeeded by Clinical Commissioning Groups (CCGs) in 2013. We used PCTs as the basis of site selection as these had a population base to derive admission rates.

Admission rates for people aged 85+ were calculated from HES data (2007/08-2009/10).(20) Data were not available for some PCTs due to mergers. For the 143/154 (93%) PCTs for which we had data, a regression co-efficient was calculated for the change in admission rates over the three-year period, adjusting for population size and age, which was used to rank PCTs.

After applying our inclusion and exclusion criteria (see below) we selected the three sites where rates of admission had increased most rapidly, and the three where they had declined most rapidly. Each site comprised an acute hospital trust, its linked PCT, the provider of community health services, and adult social care. An inclusion criterion was that more than 80% of acute admissions for people aged 85+ from the PCT were admitted to one acute Trust, so that there was at least a potential partnership between these organisations. Sites were excluded if they were known to be experiencing significant reconfiguration as reflected in publicly available information. Table 1 shows the ranking and admission rates for the selected sites: improving sites had an annual decrease in admission rates for those aged 85+ of 1-2%, and deteriorating sites an annual increase of 6%.

Quantitative data collection

After the sites had been selected we used updated HES data to examine 85+ admission rates over five years (2007/08 to 2011/12) rather than just the three years used for selection. We used the NHS portal (now NHS digital(21)) to examine admissions for acute and chronic ambulatory care sensitive conditions (ACSCs)(22) from 2007/08 to 2009/10. These are age adjusted rates per 100,000; data are not available for specific age groups. In the selected sites, invitations to participate were sent to the chief executives of the PCT and acute trust. In all cases, there was initial agreement from both parties. We then invited participation from the organisation responsible for community health services and social services.

Qualitative data collection

In each selected site, a key individual was identified in each organisation who advised on potential key informants. Further participants were identified by snowball sampling.(23) In 2013, two rounds of data collection were conducted. In the first round, an understanding of the system's history and drivers was sought in interviews with key high-level informants, including commissioners and managers of health and social care with responsibility for those aged 85+, and clinicians and care providers with leadership roles in primary care, emergency departments (ED), social care, intermediate and secondary care. In the second round, we examined specific components of the system, using in-depth interviews and focus groups with those involved in delivering care, to explore

For peer review only - http://bmjopen.bmj.com/site/about/guidelines.xhtml

issues involved in translating policy directives to changes in provision of care. These included clinicians in Emergency Departments and Acute Medical Units (AMUs), managers of intermediate and integrated care provision and clinicians in primary care. In each site, a focus group was convened including representatives of carers and service users to capture their perspectives.

Topic guides were based on the literature and agreed by the research team. The structure of the topic guide was as follows: views on unplanned admissions, views on system characteristics, specific questions on system characteristics, changes and recommendations, patient public involvement (PPI), outcomes. A detailed topic guide is presented in appendix 1.

For each site, data on population characteristics and admission rates were prepared to prompt discussion. Interviews were conducted at the workplace by research fellows (ER and KP) with substantial experience of policy focused qualitative work, who also made extensive fields notes for each site. Interviews and focus groups lasted 30-60 minutes and were recorded and transcribed verbatim. Data were collected between January 2012 and December 2013.

Analysis

Qualitative data analysis was undertaken in a stepwise, interpretative approach. Firstly, all data from each case site was assigned to individual members of the project team for initial inductive, open coding. The second and main stage of data analysis involved two independent researchers developing detailed case reports. Following a framework approach (24) all data items were systematically scrutinised with data coded according to the 7S categories. These codes and descriptions provided the basis of regular discussion amongst the multi-disciplinary research team, first for reviewing the consistency of the analysis process (inter-coder), i.e. so that similar codes related to similar phenomena; and then for debating and agreeing the thematic interpretation of data. As part of this process the similarities and differences between codes were analysed, especially when relating data the 7S model, to ensure that data/codes were categorised in ways that were sufficiently distinct, or where they shared common or complementary features they were aggregated into higher order codes, which was especially important when relating the data to the McKinsey model. NVivo software (25) was used to provide an audit trail. Guidance for coding was agreed by the team, including how items would be categorised according to the 7S framework. In line with the principle of constant comparison, each category was systematically checked for its internal consistency and inter-relationships.(26) Through the processes of analysis and interpretation the research team was especially concerned to test the reliability and confidence of interpretation through looking for counterfactuals in the data that could represent contingencies to the emerging interpretations. Illustrative and exemplar extracts of data are provided in the subsequent results section, and further empirical data can be found in the main study report.

Patient and Public Involvement

The study was presented to the Leicester older people's research PPI forum at the planning stage, and a representative from this forum was a member of the Steering Group.

For peer review only - http://bmjopen.bmj.com/site/about/guidelines.xhtml

NHS ethical approval was not required as patients were not being interviewed. Ethics approval was obtained from University of Leicester and R&D approvals from participating organisations. Informed consent was obtained from all participants.

Results

Quantitative findings

Table 2 presents data on rates of 85+ admissions between 2007/08 and 2011/12. In improving sites these increased by 3.3% (range -2.4% to 10.6%) and in deteriorating sites by 22.7% (range 16.3% to 29.2%). During the 5 year period, the mean linear regression slope per annum was 0.003 (0.3%) for improving sites and 0.022 (2.2%) for deteriorating sites. In general, rates of admission remained fairly stable in the additional two years we examined after site selection. Between using HES data for selection and this analysis, some corrections had been made to 2007/8 data.

Changes in rates of admission for ambulatory care sensitive conditions (acute conditions such as otitis media that could normally be managed without admission and chronic conditions, such as diabetes, in which disease management could prevent exacerbations needing admission (8)) are also shown in table 2. In the three years examined, rates for acute conditions fell by 8.5% in improving sites (range -17.5% to 8.3%) and increased by 18.0% in deteriorating sites (range 4.8% to 26.8%). Mean rates of change differed less for chronic conditions, but with bigger variations between sites. They reduced by 3.9% in improving sites (range -7.4% to 0.4%) and increased by 1.0% in deteriorating sites (range -14.1% to 16.0%).

Qualitative findings

In total 142 informants contributed to either individual interviews or focus groups, as shown in table 3, which also provides a brief description of the sites The number of contributions from each site varied, in part because some agencies declined to participate. In site I2, community services and social services were provided by a single organisation. The number of informants was greater in improving than deteriorating sites (91 versus 51)

Findings are presented by 7S themes, as summarised in Table 4 Quotes are identified by site and employing organisation of respondent (PCT/CCG: A; community services: B; acute trusts: C) and respondent number. All quotes presented are from one to one interviews.

1. Strategy

This was defined as the plan of activity for the whole system, and alignment of the system to its goals. Improving sites exhibited more of a shared and comprehensive system-wide strategy for managing unplanned care, including specific policies and procedures for older people. These strategies and policies were shared across the wider health and social care system suggesting an underlying basis of collaboration and coordination, and a reduced risk of system dominance by one provider.

For peer review only - http://bmjopen.bmj.com/site/about/guidelines.xhtml

We had an audacious programme goal, which was all about reducing emergency admissions, and we had quite a lot of buy-in … to a whole-system approach. I1 A-01 (commissioning manager, CCG)

For the whole time I've been here, it (urgent care) has been a top priority. We want urgent care pathways (including ambulance, ED and admissions) to be as high quality as possible. I3-A-02 (commissioning manager, CCG)

Deteriorating sites revealed less evidence of a system strategy. Although individual system components had developed strategies for aspects of unplanned care, such as reducing length of stay, there was less appreciation of how the components of the wider health system should work together. Strategies tended to be dominated by acute care provision to the detriment of policies to expand primary and community care.

The system plans have bullets like 'we'll support care close to home but we'll support financial sustainability of the acute hospitals.' Unless you have some sort of integration, those two things are mutually exclusive in the long term. D1-A-03 (service redesign manager, CCG)

An important difference between improving and deteriorating sites was their approach to improvement projects. In improving sites, these were generally well-resourced, often through funding arrangements linked to national initiatives. Moreover, they were usually given time to develop and embed into practice, rather than being subject to changing fashions or emerging policies. In contrast, in deteriorating sites projects tended to be more reactive and short-lived with little follow through.

We've piloted lots of good things, but it's been the usual story of just doing pilots and not doing them at sufficient scale, we've dabbled in things and haven't really followed them sufficiently through. D3-A-03 (commissioning director, CCG)

2. Structure

This was defined as how different components of the system related to each other. In improving sites there was closer integration of primary, acute and community services. This was facilitated by fewer organisations providing services and clearer geographical boundaries.

X was one of the few dedicated Community Trusts in the country...there's not several different providers. Patients can't get moved around the system because there's only basically GP practices, intermediate tier and one acute trust. I1-B-01 (rehabilitation manager, community trust)

The care trust... was integrated – the health organisation held the social care budget on behalf of the council ... that alone meant that the approach to commissioning was truly integrated at the budget and organisational level. So that I think is fundamental. I2-A-01 (service lead, unplanned care, CCG)

For peer review only - http://bmjopen.bmj.com/site/about/guidelines.xhtml

We hardly have any cross-boundary issues, it's just one social services department, one acute trust, one community trust, one mental health trust and one health commissioner – that's it. I3-A-02 (commissioning manager, CCG)

In deteriorating sites, there was less evidence of integration between acute, primary and community services. Governance and funding arrangements were more complex, with different ways of working. There was often emphasis on key care stages, such as admission and discharge, but not on the wider constellation of agencies, handovers and transitions that patients face across the system.

The services are so complex that we as health professionals find it difficult and I think … for somebody who is over 85 or for anybody I think - it's really difficult for them to understand where to go for help and I think so by default people know that this (A and E) is open 24 hours a day, you can literally just walk in and you'll be helped. D1-C-01 (service coordinator for older people, acute trust)

I don't think it [unplanned admissions], is managed very well really in this area which is one of the reasons why you see relatively high admission rates. Part of the reason for that is because there are separate organisations each with their own agendas and each with their own pressures.D2-C-01 (clinical director, unscheduled care, acute trust)

3. Systems

This was defined as individual services contributing to the whole system. The most consistent differences between improving and deteriorating sites were in GP services, intermediate care and ED provision.

General practice

In improving sites, general practice appeared better supported financially, more innovative, for example with IT systems, and better integrated with other providers. There was closer alignment of out-of-hours GP services with either community or acute NHS providers, which facilitated closer integration of primary, acute and community services, especially for information sharing and continuity of care.

Round here I think GPs are more proactive in managing their patients. If you're a GMS [General Medical Services] practice there's no incentive for you keeping your admission. We had our PBC [practice-based commissioning] budgets and if we were within our budget there'd be some financial reward for that. I2-A-01 (service lead, unplanned care, CCG)

(IT system) facilitates three-way conversations; the GP will be on the line, the nurse would be on the line and the acute physician from AMU [the acute medical unit] would be on the line and having a conference about whether or not it's appropriate that person ought to go to hospital. I1-A-01 (commissioning manager, CCG)

In deteriorating sites there was a sense of under-investment and insufficient planning for primary care. GP practices were seen as providing a more limited set of services with problems of access due in part to single handed practices and half day closures, thereby increasing demand on Emergency

For peer review only - http://bmjopen.bmj.com/site/about/guidelines.xhtml

Departments (EDs). Changes in out of hours GP provision was also thought to impact on ED attendances.

There's been underinvestment in primary care, there hasn't been a clear primary care strategy… there's been underinvestment in primary care local enhanced services compared to other places. D3-A-03 (commissioning director, CCG)

We've got a high proportion of (older) GPs, it's I think over 50% who have retired, taken their pension and come back. So there is no motivation for them to change at all. D1-A-02 (head of development, CCG)

Intermediate Care

Intermediate care provision at scale was widely seen to be important. This was more developed at improving sites but even here, short term funding meant services were not stable. Although type of provision varied, for example the balance of home and institutionally based provision, key elements included a single point of access, a unified system often from a single provider, and multidisciplinary working to reduce duplication.

We have a single point of access manned by advice officers, behind which there are three levels of triage– at every level of triage it's an integrated triage between health and social care.I2-A-03 (strategic adviser, adult social care)

The multidisciplinary teams in intermediate care have made a difference– you know, sometimes, one person's seeing a physio, an OT and a nurse, and they just need to see one member of the team. I1- B-02 (head of reablement, community trust)

Emergency Departments

There was agreement that skill mix in ED could influence the number of older patients admitted. Initiatives in improving sites included provision of geriatricians in ED, more senior staffing and involvement of GPs. Linked to this were initiatives to provide GP with accessible alternatives to acute admission, such as rapid access clinics or telephone consultations with a geriatrician.

GPs in A & E – well GPs have been in the hospital ever since I've been here, so they've either been co-located, so very close to A & E or in A & E. I2-A-02 (commissioning manager, CCG)

We introduced our (older people's) clinic. If they (GPs) have got an elderly patient with them in the surgery, they're just not sure what to do, they just pick up this phone line, and there's a geriatrician who will advise them. I3-C-04 (director, acute and critical care, acute trust)

4. Shared values

Shared values within and between components of the system appeared important. Improving sites were characterised by stable staffing and leadership that supported continuity of purpose, fostered trust and collaborative working, and maintained commitment to improvement. In contrast,

For peer review only - http://bmjopen.bmj.com/site/about/guidelines.xhtml
For peer review only

deteriorating sites had higher staff turnover and appeared more distracted by short term changes in policy.

> *It (stability) helps in terms of building those relationships and building trust and allowing us to perhaps take more risks. I2-A-02 (commissioning manager, CCG)*

> *I think the things that work really well are the relationships at an operational, and to a degree going into strategic work, and I think those individual relationships, and people knowing each other and having a level of trust. I3-C-02 (commissioning manager, CCG)*

> *No-one's in place long enough … the natural political cycle is shorter than the natural planning cycle for the health system. D3-A-01 (GP, CCG board member)*

Several other values emerged from the interviews. These included a general belief that admission can be counterproductive for older people and the need to challenge a mind-set that admission is the default option.

> *Acute hospitals are fundamentally not the right place for over eighty fives.. you're far better in your home environment or in a supported environment that's not hospital..D3-C-01 (chief executive, acute trust)*

> *Well, there is a culture of admission in this hospital –someone comes to the front door, they see a junior doctor, admit them. I mean, you know, if I go on a post-take ward round and see a patient that's been admitted to one of these wards on the same day and say they can be discharged, the nurse looks at me as though I'm some sort of idiot: 'What are you talking about discharge? I haven't finished admitting them yet.' D1-C-02 (clinical director, acute trust)*

Several sites described a clash of values between commissioners, managers and clinicians, particularly when managing older people, and the importance of clinical leadership.

> *No, there's nobody clinical, there's nobody caring, there's nobody who actually does the business of looking after people, particularly the frail elderly, who are messy and don't fit into a clear protocol.I2-A-05 (GP, CCG board member)*

> *I think what we probably need now is clinically led provider organisations cause you've still got managerial led provider organisations. The doctors do talk to each other but then they don't talk to the managers and the managers talk about a different thing so it's not necessarily connected. D1-A-02 (head of development, CCG)*

Although the remaining 7S categories (Skills, Style, Staff) are shown separately in Table 4, we felt it was clearer to present findings in these categories in the context of the four categories presented above. For example, there was overlap between style and values, and issues of skills and staff were often raised in the context of specific system, for example ED.

For peer review only - http://bmjopen.bmj.com/site/about/guidelines.xhtml

Discussion

Summary of findings

We found some important differences between sites in which admission rates for people aged 85+ had increased most rapidly and sites in which these rates had stabilised or declined. In improving sites there was more evidence of strong strategic leadership, enabling the development of a comprehensive system-wide strategy, including specific policies and procedures for older people, which were shared across a more integrated health and social care setting. This encouraged longer-term, consistent development of strategies, often in the face of changing national imperatives. This stability also allowed trust and shared commitment to be established and the emergence of common values across the system.

In improving sites, primary care appeared stronger, both in terms of service provision and strategic engagement. This could be one reason why admissions for ambulatory care sensitive conditions fell on average in improving sites and increased in deteriorating sites. There is also evidence presented in our funder's report (18) that GP access was better in improving sites. Intermediate care was also more developed in improving sites. These services appeared to work best when provided at scale and fully integrated with each other, offering round-the-clock availability with a single point of access, shared information systems and specialist nursing and geriatric support. Improving sites also seemed more equipped to reduce admissions of older people from ED, through a variety of initiatives, including more senior staffing, involvement of GPs and provision of specialist nurses or geriatricians. In summary, improving sites generally made fuller use of a suite of strategies to reduce unplanned admissions, and importantly had a more system-wide outlook and strategic approach.

Comparison with previous studies

Findings from several recent qualitative and mixed methods studies are consistent with those reported here. A study examining six emergency and urgent care sites suggested that improving GP out of hours access, senior review in ED and multidisciplinary teams could reduce admissions(27), and others have emphasised how the culture and staffing in ED can influence admission rates (28, 29). We are not aware of previous qualitative work examining the whole system, but our findings support recommendations on integrated care made by the Kings Fund, including 'sharing sovereignty', developing a persuasive vision and establishing leadership.(30). More recently a CQC report concluded: 'To truly coordinate care, local system leaders must ensure there is a golden thread linking vision to delivery, so that everyone involved can not only share the vision but see themselves as part of the team that delivers it'.(31)

Strengths and limitations

Our study design offered strong internal validity through in-depth analysis within case and structured comparison between cases.(32) The design and conduct of this study reflects a number of features to enhance trustworthiness.(33). The approach allowed a common method of data collection and analysis subject to open scrutiny by the wider research team and advisors, enhancing

For peer review only - http://bmjopen.bmj.com/site/about/guidelines.xhtml

dependability. In terms of confirmability, the researchers were supported in being reflexive about their own role in data collection, including review meetings with the wider research team.

That researchers and participants knew how each site had performed helped to inform the interviews; by presenting data on admissions we were able to engage in a more detailed discussion of issues and strategies. It is unlikely that informants would be unaware of their own performance. However this approach may have meant that both interviewers and participants may have focused on what was perceived to be working well in improving sites, and on more negative issues in deteriorating sites. In retrospect, blinding some rounds of data analysis to categorisation of sites may have reduced the risk of bias.

The study had several other limitations. Our selection criteria were based on changes in historic data; the additional data on admissions for 2010/11 and 2011/12 showed that over the five year period differences between improving and deteriorating sites persisted but in most sites stabilised somewhat over the last two years, and the trajectory of improvement or deterioration slowed. As with any study of outliers there is a possibility that some changes represented regression to the mean. Furthermore there were differences in performance within improving and deteriorating groups: in sites I1 and I3 the five year trend showed a small increase in admission rates, although less than in deteriorating sites. Some informants found it challenging to reflect on past events rather than the current situation. The strongest interview data came from informants who had been in post for the period of interest and so able to provide an institutional memory. This problem was compounded by the fact the study was conducted during a period of organisational upheaval in the NHS, leading to many informants being relatively new in post. Finally, the snowball sampling technique may have led to under-representation of some groups, such as ambulance services.

The pace of change within the NHS has quickened since the study was conducted, with additional challenges imposed by austerity. However, we feel our key messages remain relevant as they emphasise the need for strategies and stability that can weather the impact of ongoing changes. We also acknowledge that sites with a more complex mix of acute trusts (which we deliberately excluded) may experience different or additional problems and solutions.

The McKinsey 7S framework was effective in enabling the systematic investigation of system components and their interaction, but was less useful in mapping more abstract issues such as style and values. The framework appeared better suited to examining individual organisations, rather than large complex systems of inter-dependent heterogeneous system actors, which may have been further understood and analysed using, for example, Beer's Viable Systems Model (22).

Implications for policy makers and service providers

This study supports taking a whole system approach in designing services for older people. This is best achieved by developing a sustained and shared vision focusing on outcomes that matter to older people(12), establishing strong leadership without dominance by one organisation, maximising integration and minimising complexity within the system. These findings have potential to inform the practical implementation in England of Strategic Transformation Partnerships and Accountable Care Organisations, vehicles designed to promote system-based collaboration. (34)

For peer review only - http://bmjopen.bmj.com/site/about/guidelines.xhtml

Conclusions

Health and social care communities which attenuated unplanned admissions of people aged 85+ prioritised developing a shared vision and strategy, encompassing multiple organisations and backed by strong leadership and shared values. This allowed sustained implementation of a suite of interventions, including better-developed primary and intermediate care services working closely with the hospital and emergency departments.

Data sharing

Anonymised transcripts are available from the corresponding author.

Contributions

All authors contributed to study design, and analysis, and have approved the final manuscript. Andrew Wilson led the study, Richard Baker and Stephen Rogers contributed expertise in primary care and quality improvement, John Bankart and Stoyan Kurtev led on selection of sites. Jay Banerjee and Simon Conroy constructed expertise on ED policies and geriatric medicine respectively. Ran Bhamra contributed expertise on system theory and Justin Waring led the analysis. Kay Phelps and Emma Regen led on fields work and conducted most of the interviews.

Acknowledgements

We are grateful for the support of members of the steering group throughout the project, and for advice from the Leicestershire, Northamptonshire and Rutland Older People's Research Patient and Public Involvement Forum. Access to sites was facilitated by the NIHR Clinical Research network. Most of all we would like to acknowledge participants at all study sites for their time and the candid insights they provided.

Conflict of interest

The authors declared no potential conflicts of interest with respect to the research, authorship and/or publication of this article.

Funding

The project was funded by the NIHR Health Service and Delivery and Research Programme (project number 10/1010/05). The views and opinions expressed in this article are those of the authors and do not necessarily reflect those of the Health Service and Delivery and Research Programme, NIHR, NHS or the Department of Health.

For peer review only - http://bmjopen.bmj.com/site/about/guidelines.xhtml

References

1. Bobrovitz N, Onakpoya I, Roberts N, Heneghan C, Mahtani KR. Protocol for an overview of systematic reviews of interventions to reduce unscheduled hospital admissions among adults. BMJ open. 2015;5(8):e008269-e.
2. García-Pérez L, Linertová R, Lorenzo-Riera A, Vázquez-Díaz JR, Duque-González B, Sarría-Santamera A. Risk factors for hospital readmissions in elderly patients: a systematic review. QJM: An International Journal of Medicine. 2011;104(8):639-51.
3. Tang N, Stein J, Hsia RY, Maselli JH, Gonzales R. Trends and Characteristics of US Emergency Department Visits, 1997-2007. JAMA. 2010;304(6):664-70.
4. Schuur JD, Venkatesh AK. The Growing Role of Emergency Departments in Hospital Admissions. New England Journal of Medicine. 2012;367(5):391-3.
5. Raphael Wittenberg LS, Barry McCormick, Jeremy Hurst. Understanding emergency hospital admissions of older people. Oxford: 2014.
6. Purdy S. Avoiding hospital admissions - What does the research evidence say? London: Kings Fund, 2010.
7. University of Birmingham Health Services Management Centre. Reducing Unplanned Hospital Admissions: what does the literature tell us? Birmingham: 2006.
8. Tian Y, Dixon A, Gao H. Emergency hospital admissions for ambulatory care sensitive condtions: identifying the potential for reducations. London: The Kings Fund, 2012.
9. O'Cathain A, Knowles E, Maheswaran R, Pearson T, Turner J, Hirst E, et al. A system-wide approach to explaining variation in potentially avoidable emergency admissions: national ecological study. BMJ Quality & Safety. 2014;23(1):47-55.
10. Sager MA, Franke T, Inouye SK, Landefeld CS, Morgan TM, Rudberg MA, et al. Functional Outcomes of Acute Medical Illness and Hospitalization in Older Persons. Arch Intern Med. 1996;156(6):645-52.
11. Landeiro F, Roberts K, Gray AM, Leal J. Delayed Hospital Discharges of Older Patients: A Systematic Review on Prevalence and Costs. The Gerontologist. 2017:gnx028.
12. Akpan A, Roberts C, Bandeen-Roche K, Batty B, Bausewein C, Bell D, et al. Standard set of health outcome measures for older persons. BMC Geriatrics. 2018;18(1):36.
13. Shepperd S, Iliffe S, Doll H, Clarke M, Kalra L, Wilson AD, et al. Admission avoidance hospital at home. Cochrane Database of Systematic Reviews [Internet]. 2016; (9). Available from: http://onlinelibrary.wiley.com/doi/10.1002/14651858.CD007491.pub2/abstract;jsessionid=D3133E6EEEA6E31B6A45F33C66800223.f03t02
14. NHS Benchmarking Network. National Audit of Intermediate Care. Manchester: NHS Benchmarking Network, 2013.
15. Barker I, Steventon A, Deeny SR. Association between continuity of care in general practice and hospital admissions for ambulatory care sensitive conditions: cross sectional study of routinely collected, person level data. BMJ. 2017;356.
16. White AL, Armstrong PAR, Thakore S. Impact of senior clinical review on patient disposition from the emergency department. Emergency Medicine Journal. 2010;27(4):262-5.
17. Damery S, Flanagan S, Combes G. Does integrated care reduce hospital activity for patients with chronic diseases? An umbrella review of systematic reviews. BMJ Open. 2016;6(11).
18. Wilson A, Baker R, Bankart J, Banerjee J, Bhamra R, Conroy S, et al. Establishing and implementing best practice to reduce unplanned admissions in those 85 years and over through system change (Establishing System Change for Admissions of People 85+(ESCAPE 85+)): a mixed-methods case study approach. Health Services and Delivery Research. 2015;3:37.
19. Yin RK. Multiple case studies with embedded sub cases. Case Study Research: Design and Methods. Fourth Edition ed. California: Sage Publications; 2009.
20. NHS_Digital. Hospital Episode Statistics.
21. NHS_digital. Emergency hospital admissions: acute conditions usually managed in primary care: indirectly standardised rate, all ages, annual trend, F,M,P 2016 [cited 15/05/2018]. Available

For peer review only - http://bmjopen.bmj.com/site/about/guidelines.xhtml

from: https://digital.nhs.uk/data-and-information/publications/clinical-indicators/compendium-of-population-health-indicators/compendium-hospital-care/current/emergency-admissions/emergency-hospital-admissions-acute-conditions-usually-managed-in-primary-care-indirectly-standardised-rate-all-ages-annual-trend-f-m-p.

22. Purdy S, Griffin T, Salisbury C, Sharp D. Ambulatory care sensitive conditions: terminology and disease coding need to be more specific to aid policy makers and clinicians. Public Health. 2009;123(2):169-73.

23. Marshall MN. The key informant technique. Family Practice. 1996;13(1):92-7.

24. Gale N, Heath G, Cameron E, Rashid S, Redwood S. Using the framework method for the analysis of qualitative data in multi-disciplinary health research. BMC Medical Research Methodology. 2013;13(1):117.

25. NVivo qualitative data analysis software. QSR International Pty Ltd Version 7. 2006.

26. Boeije H. A purposeful approach to the constant comparative method in the analysis of qualitative interviews. Qual Quant. 2002;36(4):391 - 409.

27. O'Cathain A, Knowles E, Turner J, Hirst E, Goodacre S, Nicholl J. Variation in avoidable emergency admissions: multiple case studies of emergency and urgent care systems. Journal of Health Services Research & Policy. 2016;21(1):5-14.

28. Pinkney J RS, Benger J, Brant H, Joel-Edgar S, Swancutt D, et al. How can frontline expertise and new models of care best contribute to safely reducing avoidable acute admissions?

A mixed-methods study of four acute hospitals. Health Serv Deliv Res. 2016;4(3).

29. Pope I, Burn H, Ismail SA, Harris T, McCoy D. A qualitative study exploring the factors influencing admission to hospital from the emergency department. BMJ Open. 2017;7(8).

30. Ham C, Walsh N. Making integrated care happen at scale and pace. London: The Kings Fund; 2013.

31. Commission CQ. The state of health care and adult social care in England 2016/17. London: Care Quality Commission 2017.

32. Mintzberg H, Lampel JB, Quinn J, Ghoshal S. The Strategy Process: Concepts, Contexts, Cases, 5/E. Harlow: Pearson; 2014.

33. Lincoln YS, Guba EG. Establishing trustworthiness. London: Sage; 1985. 289-331 p.

34. NHS_Providers. STPs and accountable care: background briefing. 2018.

For peer review only

For peer review only - http://bmjopen.bmj.com/site/about/guidelines.xhtml

PCT<br>Rank for slope (n=143)	85+ admission rate (number of admissions/100 population aged 85+)			Slope (per annum change)	% change in rate	% admissions to linked hospital trust	% aged 85+	Reference in paper
	2007/08	2008/09	2009/10					
4	0.55	0.51	0.51	-0.02	-7.3	89	2.6	I1
5	0.61	0.6	0.57	-0.02	-6.6	87	2.6	I3
9	0.41	0.41	0.39	-0.01	-4.9	83	2.2	I2
132	0.48	0.54	0.59	0.06	22.9	92	2.2	D1
133	0.41	0.45	0.52	0.06	26.8	87	1.7	D3
135	0.49	0.59	0.61	0.06	25.5	83	1.8	D2

Table 1: Selection of improving (I) and deteriorating (D) Primary Care Trusts (PCT)

For peer review only

For peer review only - http://bmjopen.bmj.com/site/about/guidelines.xhtml

	I1	I2	I3	D1	D2	D3		Improving sites average *	Deteriorating sites average *
**85+ admissions/100 aged 85+ per annum**									
2007/08	0.47	0.41	0.57	0.48	0.49	0.40		0.48	0.46
2008/09	0.51	0.42	0.6	0.55	0.59	0.45		0.51	0.53
2009/10	0.51	0.40	0.58	0.60	0.61	0.53		0.50	0.58
2010/11	0.54	0.39	0.58	0.60	0.56	0.48		0.50	0.55
2011/12	0.52	0.40	0.58	0.62	0.57	0.49		0.50	0.56
% change between 2007/8 and 2011/12	10.64	-2.44	1.75	29.17	16.33	22.50		3.32	22.67
Linear regression slope (per annum change in rate)	0.013	-0.005	0	0.033	0.013	0.020		0.003	0.022
**Acute ACSC all ages. Indirectly age and sex standardised rate per 100,000**									
2007/08	424	579	462	367	397	619		488	461
2008/09	515	591	347	388	461	699		484	516
2009/10	350	627	387	449	416	785		455	550
% change between 2007/8 and 2009/10	-17.45	8.29	-16.23	22.34	4.79	26.82		-8.46	17.98
Linear regression slope (per annum change in rate)	-37	24	-37.5	41	9.5	83		-16.83	44.50
**Chronic ACSC all ages. Indirectly age and sex standardised rate per 100,000**									
07/08	220	240	148	188	177	247		203	204
08/09	276	247	104	212	177	265		209	218
09/10	210	241	137	218	152	249		196	206
% change between 2007/8 and 2009/10	-4.55	0.42	-7.43	15.96	-14.12	0.81		-3.85	0.88
Linear regression slope (per annum change in rate)	-5	0.5	-5.5	15	-12.5	1		-3.33	1.17

Table 2: Changes in admission rates for patients aged 85+, and rates of admission for acute and chronic ambulatory care sensitive conditions (ACSC) in improving and deteriorating sites

*Averages derived from rows not columns.

For peer review only - http://bmjopen.bmj.com/site/about/guidelines.xhtml

Sites		Description					Participants					
		ONS classification	Ranking of population size (151 PCTs, 1 = largest)	N (%) aged 85+	Deprivation rank (151 PCTs, 1 = most deprived)	Acute provision	Acute Trust	PCT/ CCG	Community Services	Social services	PPI	Total
I1	Major city	Regional centre	85	6527 (2.6)	56	1 university hospital, 1 district general hospital	declined	2	13 (7 individual, 2 focus groups [n=2 and 4])	declined		15
I2	largely rural area comprising three small to medium-sized towns.	Manufacturing town	139	3546 (2.6)	40	3 district general hospitals	6	6	16 (7 individual, 2 focus groups [n=9])		Focus group (n= 5)	33
I3	semirural and urban conurbation in close proximity to metropolitan area.	Industrial hinterlands	42	7970 (2.2)	50	2 district general hospitals	5	3	24 (4 individual, 3 focus groups [n=6,6,8])	2	Focus group (n=9)	43

19

For peer review only

D1	Major city	Centre with industry	56	6667 (2.2)	43	1 large acute hospital	7	3	3	2	Focus group (n=5)	20
D2	three small to medium-sized towns	Centre with industry	120	3703 (1.7)	22	4 district general hospitals	10	3	2	4	Focus group (n=5)	24
D3	mixed urban and rural area	New and growing town	118	3463 (1.8)	119	1 large acute hospital	2	3	declined	1	1	7
Total							30	20	58	9	25	142

Table 3: Description of sites and informants by organisational category

For peer review only - http://bmjopen.bmj.com/site/about/guidelines.xhtml

For peer review only

	7S						
	Strategy	Structure	Systems	Shared values	Skills	Style	Staff
I1	High levels of investment in community provision.<br>Early engagement of all stakeholders in strategy development	Strong linkages between hospital, GP and community care<br>Small number of large providers	Innovative cross-sectoral technology systems<br>Unified provision of intermediate care	Professionals willing to work together and bend hierarchies to reach shared goals<br>Pride in in providing services to keep older people out of hospital	Staff have perseverance and skills to see through projects and get people on board	Regular contacts between hospital and community providers	Effective multidisciplinary teams in intermediate care<br>Recognised need to involve pharmacists to reduce excessive medication
I2	Care trust developed strategy across social and community health services	Integrated budget for commissioning health and social care	Focus on practice based commissioning providing incentives for GPs to reduce admissions<br>Single point of access 24/7 for intermediate care	Strong and stable organisational cohesion	High levels of interpersonal skills enable effective working relationships	Close links between GPs and other service providers	Longstanding and close working relationships
I3	Urgent care a top strategic priority<br>Strategy recognises the importance of community care	Lack of boundary issues helps maintain a clear structure, with small number of providers	Out of hours run by through community trust<br>Rapid access to clinics and telephone consultations with geriatricians	Strong organisational cohesion	High levels of skills in community teams	Some 'blame culture' when services are pressurised	
D1	Frequent changes in leadership	Complex structures for health community care	Perception that GPs were demotivated	Historically poor relationship between	Perception that care	Culture of admission	Recognised shortage of geriatricians

For peer review only - http://bmjopen.bmj.com/site/about/guidelines.xhtml

	roles and regional strategies<br>Acute Trust dominant in determining strategy		Lack of well-functioning multi-disciplinary teams	Trusts and community providers	home staff are under skilled	from ED as default option	
D2	Lack of clear strategy on unplanned admissions; more focus on reducing length of stay than admission avoidance	Poor integration between primary and secondary care, and between ambulance services and acute Trust	Frequent restructuring of intermediate care	Lack of shared culture between organisations	Recognised need for skilled geriatricians in acute medical unit	Recent focus on clinical leadership	Lack of senior medical staff in ED<br>Recent investment in geriatricians and community matrons
D3	More focus on elective care than urgent care<br>Successful pilots not followed through	Frequent changes in structure of system, including hospital sites and structure of intermediate care	Low investment in primary care	Conflict between medical, rehabilitation and managerial values	Perception that insufficient staff have skills needed to assess frail elderly	Recent focus on clinical leadership	Inadequate provision of community matrons

Table 4: Features of sites by McKinsey 7S categories

For peer review only - http://bmjopen.bmj.com/site/about/guidelines.xhtml

Figure 1: The McKinsey 7S Framework and how it was applied

Strategy: the plan of activity for the whole system, and alignment of the system to its goals

Structure: how different components of the system related to each other.

Systems: individual services contributing to the whole system.

Shared values: the norms and standards that guide the behaviour of the human elements within the system.

Style: the style of management used by the system leadership.

Staff: training, motivation and rewards of the staff.

Skills: specific skills existing and required by staff in order to best execute their duties

For peer review only

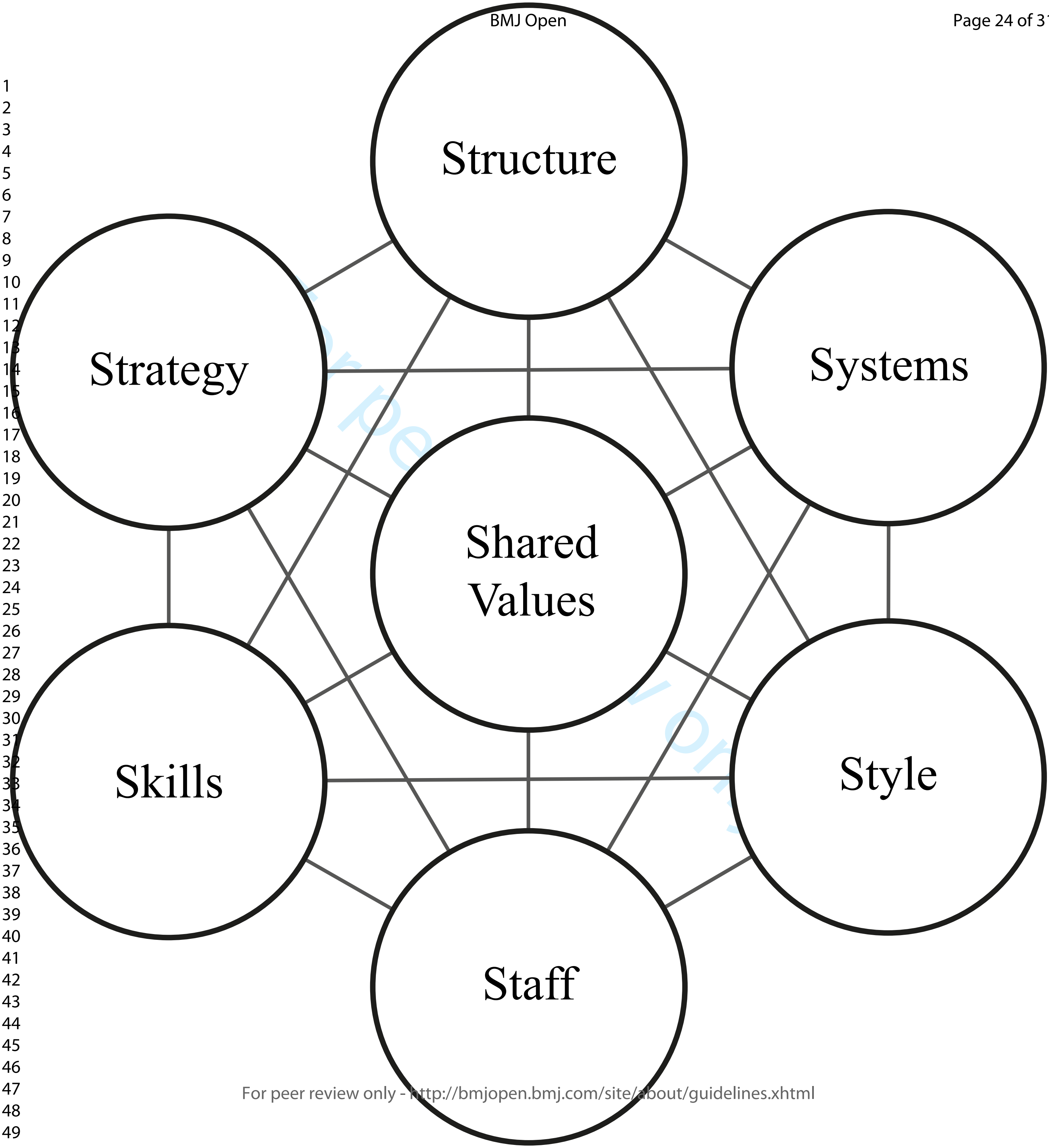

For peer review only - http://bmjopen.bmj.com/site/about/guidelines.xhtml

Appendix 1: detailed topic guide

Unplanned admissions in the area: priorities, levels and changes Ask interviewee to give a reaction to the data presented to them giving the rates of unplanned admissions of people 85+ in their area during 2005–9.

Historical perspective from 2005 onwards.

Possible questions and prompts:

(a) Were you aware of the rate of unplanned admissions to hospital of people 85+ in your area? (b) Did you know if your rates were increasing decreasing or staying the same? – What would you say were the reasons for that?

(c) Are the rates of unplanned admissions to acute hospital of people 85+ an issue in your organisation? – Do they need to be reduced?

(d) Do you collect/collate any local data on unplanned admissions and if so can we see the reports/have access to the data?

Whole system characteristics, configuration, monitoring and changes over time What are the important elements/components which you believe have the most effect on the rates of unplanned admissions of people 85+ in your area?

Possible prompts (could use star model to illustrate):

(a) Policies. – Procedures, service delivery, working practices.

(b) People, staffing, leadership, behaviour. (c) Finance and budgets. (d) Infrastructure, geography, buildings, IT. (e) Partner organisations. (f) Shared vision.

Possible questions:

(a) What characteristics are associated with changes in unplanned admissions of people 85+? (b) What changes have you made as a result of the increasing number of people over 85? (c) What changes do you think you need to make to reduce unplanned admissions of people 85+? (d) What changes are planned to reduce unplanned admissions of people 85+? (e) What have been the historical drivers of change? – Leadership. – Finance. – Targets.

(f) When making changes what have been the main challenges? (g) What have been the main outcomes of change? – Did it achieve what was hoped for?

(h) Reasons for success or failure of changes.

Policy on unplanned admissions

(a) How have/do policies in your organisation affect unplanned admissions of people aged 85+? – Historical perspective as well as current. – E.g. A&E, ED, Primary Care, out-of-hours services, Ambulance, Staffing.

(b) What have been/are your organisation's policies around unplanned admissions? – Historical perspective as well as current.

(c) Have policies around unplanned admissions been linked/coherent with the policies of other organisations in your area? – Historical perspective as well as current.

For peer review only

(d) What policy changes have you made as a result of the increasing number of people over 85? (e) Have there been/are there clear strategies/goals within your organisation around unplanned admissions of people 85+? (f) Please can you supply policy documentation on unplanned admissions of people 85+ from 2005 to the present?

Budgets/finance/commissioning of emergency care

(a) Is there a specific budget for unplanned/emergency care for people aged 85+/older people? – Historically and currently.

(b) How much is spent on emergency care for people aged 85+? – Historically and currently.

(c) How much is spent on older people compared with other areas? – Historically and currently.

(d) What are the commissioning arrangements for unplanned admissions (of people 85+) in your area? – CCGs. – PCTs. – Involvement of secondary care.

(e) How have these arrangements changed since 2005? (f) What part have financial imperatives (cost/resources) played in commissioning services for unplanned admissions? (g) What are the incentives in commissioning which affect unplanned admissions?

Partnership working between acute and community health and social care

(a) How have acute and community health and social care worked together in the area to tackle unplanned admissions and care of older people? (b) Has there been a whole systems approach to planning care or do all the organisations commission, plan and deliver service separately? E.g. – Cooperative inter organisational networks. – Sharing information and communication. – Shared protocols and procedures. – Shared policy on unplanned admissions – linked/coherent?

(c) Have there been any integrated services or is this planned? (d) What have been the incentives to a whole systems approach? (e) What have been the challenges to a whole systems approach and how does cooperation/competition pose challenges? (f) Do the health- and social care organisations in your area have clearly defined boundaries? – What are the boundaries? – Are boundaries a problem? – Different measurement criteria for success? – Different organisational cultures? – Different regulation?

(g) Outcomes of partnership/integrated planning/services? – Successes and challenges. – Reasons for success or failure. – Obtain copies of any partnership agreements.

People, leadership, managerial and clinical relationships, staffing

(a) Who has led policy and/or changes affecting unplanned admissions of people over 85 in this area? – Organisational leadership.

(b) Have there been any professional tensions, allegiances and hierarchies which affect unplanned admissions in this area? (c) Have there been any staff satisfaction surveys carried out in your organisation? – Can we have report?

(d) Have job planning and staffing changes had an impact on unplanned admissions? (e) What impact has training had on unplanned admissions?

Infrastructure and contextual factors influencing unplanned admissions of 85+

For peer review only

(a) Have there been any local infrastructure or contextual factors or pressures influencing unplanned admissions of 85+ in this area? Possible prompts: – Geography. – Buildings/facilities. – Technology/IT systems. – Transport – ambulance services. – Environmental conditions. – Local pressures.

Procedures/service delivery

(a) Are there any issues around service delivery in your area which have affected unplanned admissions of people 85+? Possible prompts: – Who delivers urgent care services in the acute sector? – Who delivers urgent care services in the community sector? – Commissioning arrangements. – Pathways of care. – Access points for urgent and emergency care. – Admission pathways. – Management of acute care. – ED configuration. – Integrated care. – Access to and responsiveness of social care. – Management of chronic conditions. – Out-of-hours provision in the community. – Ambulance service. – Discharge procedures. – IT systems (Dashboard, NHS Pathways, discharge monitoring systems, etc.). – Governance systems (emergency care networks, joint governance frameworks).

Interventions, outcomes and evaluation

(a) What interventions in the whole system have affected or changed the levels of unplanned admissions? – Intermediate care. – Integrated care. – Reablement – End of life care. – Residential/nursing home care. – Community matrons. – Alternatives to acute admission. – Discharge initiatives. – Social care initiatives. – Chronic conditions management. – Geriatrician assessment/outreach. – Hospital outreach. – Hospice care/outreach.

(b) Who delivers these services? (c) What has been the effect of these interventions on the whole system of care? (d) What is the relationship between interventions? (e) How available and accessible are alternatives to unplanned admission? – E.g. primary and social care services.

(f) Have the outcomes of the interventions been measured or evaluated? (g) What are the reasons for the success of these interventions? (h) What are the reasons for the failure of these interventions? (i) Can I have copies of any internal evaluations?

Recommendations for reducing emergency admissions

(a) What changes have been made/need to be made to reduce unplanned admissions? – Strategy. – Organisational. – Partnership. – Commissioning. – Policy. – Service delivery. – Leadership/staffing.

(b) What lessons have you learnt in trying to reduce unplanned admissions for those 85+? – What are the challenges? – What have been the successes?

PPI involvement

(a) What PPI involvement do you have in your organisation in this area? (b) What PPI involvement do you have in policy making in this area? (c) What PPI involvement do you have in commissioning in this area? (d) What PPI involvement do you have in strategy development in this area? (e) What PPI involvement do you have in system development in this area? (f) What PPI involvement do you have in leadership and decision making in this area? (g) What PPI involvement do you have in service delivery in this area? (h) What changes have resulted from PPI involvement?

Outcomes and impact for older people and carers

(a) How do you measure outcomes for older people and carers? (b) What service changes have most affected older people and carers in this area? – What has been the impact?

For peer review only

(c) What has been the impact of particular initiatives to reduce unplanned admissions? – Refer to initiatives already mentioned above. – What is the patient experience of these services?

(d) Have outcomes for older people and carers changed over the last 5 years? – For the better? – For the worse?

(e) What changes are needed to improve emergency services and outcomes for older people 85+ and carers? (f) What are the benefits to older people and their carers of reducing unplanned admissions? (g) What are the disadvantages to older people and their carers of reducing unplanned admissions? (h) Have you carried out any patient satisfaction surveys in emergency/unplanned/urgent care in the last 5 years? – Can we have the report?

(i) Have you carried out any service evaluation looking at urgent care services for people over 85?

For peer review only

Consolidated criteria for reporting qualitative studies (COREQ): 32-item checklist

Developed from:
Tong A, Sainsbury P, Craig J. Consolidated criteria for reporting qualitative research (COREQ): a 32-item checklist for interviews and focus groups. *International Journal for Quality in Health Care*. 2007. Volume 19, Number 6: pp. 349 – 357

**No. Item**	**Guide questions/description**	**Reported on Page #**
**Domain 1: Research team and reflexivity**		
Personal Characteristics		
1. Inter viewer/facilitator	Which author/s conducted the inter view or focus group?	4
2. Credentials	What were the researcher's credentials? E.g. PhD, MD	4
3. Occupation	What was their occupation at the time of the study?	4
4. Gender	Was the researcher male or female?	Author list
5. Experience and training	What experience or training did the researcher have?	4
Relationship with participants		
6. Relationship established	Was a relationship established prior to study commencement?	no
7. Participant knowledge of the interviewer	What did the participants know about the researcher? e.g. personal goals, reasons for doing the research	no
8. Interviewer characteristics	What characteristics were reported about the inter viewer/facilitator? e.g. Bias, assumptions, reasons and interests in the research topic	no

For peer review only

**Domain 2: study design**		
Theoretical framework		
9. Methodological orientation and Theory	What methodological orientation was stated to underpin the study? e.g. grounded theory, discourse analysis, ethnography, phenomenology, content analysis	3
Participant selection		
10. Sampling	How were participants selected? e.g. purposive, convenience, consecutive, snowball	3
11. Method of approach	How were participants approached? e.g. face-to-face, telephone, mail, email	3
12. Sample size	How many participants were in the study?	4
13. Non-participation	How many people refused to participate or dropped out? Reasons?	4
Setting		
14. Setting of data collection	Where was the data collected? e.g. home, clinic, workplace	4
15. Presence of non-participants	Was anyone else present besides the participants and researchers?	no
16. Description of sample	What are the important characteristics of the sample? e.g. demographic data, date	Table 2
Data collection		
17. Interview guide	Were questions, prompts, guides provided by the authors? Was it pilot tested?	4
18. Repeat interviews	Were repeat inter views carried out? If yes, how many?	no
19. Audio/visual recording	Did the research use audio or visual recording to collect the data?	4
20. Field notes	Were field notes made during and/or after the inter view or focus group?	4
21. Duration	What was the duration of the inter views or focus group?	4
22. Data saturation	Was data saturation discussed?	
23. Transcripts returned	Were transcripts returned to participants for comment and/or correction?	no
**Domain 3: analysis and findings**		
Data analysis		

For peer review only

24. Number of data coders	How many data coders coded the data?	4
25. Description of the coding tree	Did authors provide a description of the coding tree?	4
26. Derivation of themes	Were themes identified in advance or derived from the data?	4
27. Software	What software, if applicable, was used to manage the data?	4
28. Participant checking	Did participants provide feedback on the findings?	no
Reporting		
29. Quotations presented	Were participant quotations presented to illustrate the themes/findings? Was each quotation identified? e.g. participant number	throughout
30. Data and findings consistent	Was there consistency between the data presented and the findings?	throughout
31. Clarity of major themes	Were major themes clearly presented in the findings?	throughout
32. Clarity of minor themes	Is there a description of diverse cases or discussion of minor themes?	Minor themes

For peer review only
